# Daily Multi‐Leaf Collimator positional error prediction using machine learning: A Dynalog‐based approach for patient specific quality assurance

**DOI:** 10.1002/acm2.70882

**Published:** 2026-10-27

**Authors:** Samira Aghaei Akizi, Seyed Mahmoud Reza Aghamiri, Zahra Siavashpour, Reza Ghaderi, Soharb Barimani

**Affiliations:** ^1^ Department of Medical Radiation Engineering Shahid Beheshti University Tehran Iran; ^2^ Department of Radiation Oncology Cancer Institute School of Medicine Tehran University of Medical Sciences Tehran Iran; ^3^ Clinical Research Development Unit, Shohada‐e Tajrish Hospital Shahid Beheshti University of Medical Sciences Tehran Iran; ^4^ Department of Electrical Engineering Shahid Beheshti University Tehran Iran; ^5^ Department of Biomedical Engineering Amirkabir University of Technology Tehran Iran

**Keywords:** daily dosimetric analysis in IMRT, daily MLC positional errors, machine learning

## Abstract

**Background:**

Accurate positioning of multi‐leaf collimators (MLCs) is critical for ensuring the dosimetric precision of intensity‐modulated radiation therapy (IMRT). Nonetheless, daily MLC positional variations can degrade treatment accuracy, emphasizing the need for robust methodologies to detect and predict these deviations.

**Purpose:**

This study proposes a machine learning (ML) approach to predict daily MLC positional deviations using factors contributing to positional errors, enabling daily patient‐specific quality assurance (PSQA) to assess the dosimetric impact of these discrepancies on patient treatment.

**Methods:**

DICOM‐RT and Dynalog files from 29 IMRT patients were collected, and 78 features were extracted, including aperture‐based features, mechanical parameters, and MLC check data. A LightGBM machine learning model was constructed to predict daily positional errors between planned and delivered MLC positions at each control point. For dosimetric analysis, nine independent IMRT plans were evaluated using dose volume histogram (DVH) parameters such as D_98%_, and V_100%_ for targets and D_max_, D_mean_ for Organs at Risk (OARs). Moreover, the Gamma Pass Rate (GPR) was calculated for both the delivered and predicted dose distributions, with regarding the primary planned dose distribution as the reference.

**Results:**

The proposed LightGBM model demonstrated accurate predictions, achieving Mean Absolute Error (MAE) and Root Mean Square Error (RMSE) of 0.035 mm and 0.078 mm for the training dataset, 0.070 mm and 0.164 mm for the validation dataset, and 0.054 mm and 0.144 mm for the test dataset, respectively. Leaf velocity, with a Spearman correlation coefficient of 0.703, was the most influential feature. Evaluation on nine independent clinical cases demonstrated good agreement between delivered and predicted dosimetric deviations from the planned values, with mean differences below 0.200 Gy and approximately 1.5% for target structures, and even smaller discrepancies for OARs. The GPR (3%/3 mm criterion) was about 100% for delivered doses and 99.99% for predicted doses.

**Conclusions:**

The proposed ML‐based LightGBM model offers an efficient daily PSQA tool, with the potential to enhance the treatment accuracy and support proactive clinical decision‐making in IMRT.

## 1 INTRODUCTION

Intensity‐modulated radiation therapy (IMRT) utilizes multi‐leaf collimators (MLCs) to generate a non‐uniform intensity distribution, thereby delivering highly conformal dose distributions to tumors while minimizing radiation exposure to surrounding healthy tissues.^1^, ^2^ The treatment planning system (TPS) plays an important role in the design of small, irregular segments; however, such complexity can introduce uncertainties in dose calculation.^3^, ^4^ Moreover, the mechanical complexity of the IMRT delivery mechanism may amplify operational variabilities in linear accelerators (Linacs), resulting in discrepancies between the intended and the delivered dose distributions.

To address this challenge, several reports and guidelines issued by the American Association of Physicists in Medicine (AAPM), including Task Groups 109, 119, and 218, strongly recommend the implementation of pre‐treatment patient‐specific quality assurance (PSQA).^5^, ^6^, ^7^, ^8^ PSQA is commonly performed using Electronic Portal Imaging Devices (EPIDs) and phantoms specifically designed to be integrated with one or more two‐dimensional array detectors.^9^, ^10^ The gamma index is the most frequently used metric to evaluate PSQA results, typically assessed using criteria of 2–3% dose difference (DD), 2–3 mm distance‐to‐agreement (DTA), and a Gamma Pass Rate (GPR) threshold of 95–98% to ensure reliable dose delivery accuracy.^11^

However, the PSQA is carried out exclusively after the treatment plan has been approved. In cases where the procedure fails, it may necessitate replanning and reevaluation, which can render PSQA a time‐consuming, labor‐intensive, and costly endeavor. This challenge is particularly pronounced in centers with a high workflow, as it imposes an additional burden on the staff. Identifying the sources of failure can be tricky,^12^, ^13^, ^14^ having weak correlations observed with variations in dose‐volume histograms (DVHs).^15^, ^16^ Furthermore, PSQA is usually performed just once before treatment, potentially overlooking system errors that may arise during actual treatment sessions.

Modern alternatives to pre‐treatment phantom‐based PSQA include computational software^17^, ^18^, ^19^ and machine log file analysis,^19^, ^20^, ^21^, ^22^, ^23^, ^24^, ^25^ which have been introduced to tackle the limitations of traditional PSQA methods. Although independent and computational‐based approaches can reduce specific issues associated with primary treatment planning system algorithms, they do not entirely replace conventional methods.

Machine log files, which encompass information related to treatment plans and their delivered treatment, including the mechanical positions of the gantry, collimator, jaws, and leaves, facilitate comparisons between planned and daily dose distributions. Although log file analysis mitigates certain drawbacks of direct measurement, ensuring data transfer integrity and the accuracy of linear accelerator performance, it still requires loading pre‐treatment plans, imposing a comparable burden on the machine as measurements. However, this information remains unavailable until delivery completion and does not provide direct insight into the influence of planning on MLC deviations. Additionally, historical analyzes of log files intended to predict MLC failures rely on generalized plan characteristics. Consequently, they cannot effectively detect errors that may occur in specific treatment plans.^26^

The applications of machine learning (ML) and deep learning (DL) algorithms in radiation oncology represent an innovative approach to enhance treatment quality.^27^ Valdes et al. were the first researchers to employ a weighted Poisson regression model with Lasso regularization to predict GPR across various treatment sites utilizing aperture‐based features as input variables,^28^ and the model's validity was subsequently confirmed through a multi‐institutional study.^29^ In addition, several studies have explored various ML models that incorporate plan parameters, complexity metrics, and dosiomics features as inputs to predict GPR values and determine pass/fail outcomes in PSQA procedures.^30^, ^31^, ^32^ Since ML models require feature extraction using expert physicist intervention, many studies have aimed to train DL models using various inputs such as fluence maps,^33^ sagittal dose distribution,^34^ planar dose distribution from portal dosimetry,^35^ three‐dimensional dose distribution with MLC position maps,^36^ and log file delivery fluence.^37^

Despite the advantages of ML and DL models in time efficiency, automation, and reducing staff workload, ML and DL models inherit errors from the original measurement data.^38^ Additionally, these models cannot effectively illustrate the impact of individual leaf positional deviations on the three‐dimensional dose distribution delivered to the patient.^39^ Furthermore, they struggle to account for recurring inconsistencies that may arise on the treatment day, complicating the reliability of the treatment delivery process.

Previous efforts to mitigate PSQA limitations include Carlson et al., who developed ML models (Linear Regression, Random Forest, and Cubist) to directly predict deviations in MLC positions between DICOM‐RT and Dynalog files. They found that incorporating predicted MLC positions into the treatment plans increased GPR by approximately 4.17% for head and neck cases and demonstrated strong agreement between predicted and delivered DVHs.^40^ Osman et al. also explored artificial neural networks (ANNs) to predict MLC positional discrepancies.^41^ Additionally, several tree‐based ML models were evaluated to predict leaf positional errors, using features extracted from DICOM‐RT files as inputs.^24^, ^42^ Lay et al. proposed a virtual PSQA that employed tree‐based ML algorithms to assess the impact of leaf positional, using separate models for 31 IMRT plans and 85 VMAT plans. To analyze dosimetric effects, the validated model was tested on seven IMRT and ten VMAT plans, using indices such as D_99%_, D_95%_, D_1%_, V_25‐100%_, and mean dose.^43^

Nevertheless, recent studies have demonstrated that MLC positions recorded in log files may not precisely represent delivered leaf positions due to mechanical malfunctions or calibration discrepancies.^43^, ^44^, ^45^ Consequently, incorporating leaf quality control parameters into the model can enhance the accuracy of positional error assessments derived from log file data.

It is important to note that changes in the daily operation of MLC are inevitable. The performance of MLC is routinely evaluated through daily initialization checks of Varian Clinac, which explicitly assess the functionality of the MLC. Each recorded initialization parameter can impact the daily dose distribution for each patient and should be regarded as a critical feature when evaluating daily leaf positional errors. Incorporating initialization parameters into the prediction model ensures that it is based on actual measurements, derived from MLC checks, thus mitigating the limitations of log file data.

Qiu et al. evaluated the influence of spatial features and temporal variability extracted from fluence maps of 23 VMAT plans, alongside MLC‐check parameters, to forecast leaf deviations.^46^ Using the Random Forest algorithm, they predicted average deviations of control points in Dynalog files for each leaf. They confirmed that incorporating log‐file‐derived leaf positions into DICOM‐RT files caused dose deviations between 12.948 cGy and 210.235 cGy.

Despite these advances, an important gap remains in the application of machine learning to daily MLC positional error prediction. Most previous approaches have focused primarily on predicting positional errors from treatment‐plan or log‐file‐derived features, while relatively limited attention has been given to incorporating daily MLC initialization measurements that reflect the machine's condition on the treatment day. Moreover, prediction targets have often been defined as average or aggregate leaf deviations, which limits direct propagation of the predicted errors into the DICOM‐RT files for control‐point‐specific dose recalculation.^46^

In this study, we proposed a novel approach to predict daily MLC positional deviations at the time of treatment, rather than just relying on generalized pre‐treatment predictions. A dataset comprising multiple IMRT plans from different treatment sites, along with the corresponding Dynalog files from a Varian Clinac 600C and daily MLC initialization logs, was used. Three categories of features were extracted: (a) aperture‐based features, reflecting the geometric characteristics of beam segments; (b) mechanical parameters, detailing MLC movements derived from DICOM‐RT files; and (c) initialization parameters, obtained from daily quality control logs of the MLC.

These features were used to train an ML regression model designed to estimate leaf‐level positional errors for each control point. After model validation, independent clinical patient datasets were used to evaluate the dosimetric impact of predicted versus delivered MLC deviations, providing a framework for anatomy‐based daily quality assurance (QA). The study also assessed the effectiveness of the model in predicting variations in leaf positions across different treatment fractions.

## 2 MATERIAL AND METHODS

### 2.1 Dataset collection

Twenty‐nine IMRT plans including 3 prostate, 17 head and neck, and 9 brain cases were collected from the Radiation Oncology department of Shohada‐e Tajrish Hospital, Tehran, Iran. All treatment plans were approved and created using the Eclipse TPS (version 13.0.33, Varian Medical Systems, Palo Alto, CA), with the Analytical Anisotropic Algorithm (AAA) dose calculation algorithm. The DICOM‐RT files were exported from TPS, and the corresponding Dynalog files, along with initialization parameters, were recorded during patient treatment sessions using a Clinac 600C linear accelerator equipped with a Millennium 80‐leaf MLC (two banks of 40 tungsten alloy leaves with 1 cm width at isocenter) from September 2022 to October 2022.

The dataset comprised 34,448 control points extracted from these plans/patients across the aforementioned anatomical sites. When this number is multiplied by the number of leaves, a total of 2,755,840 input samples is obtained.

### 2.2 Aperture‐based features, leaf mechanical features, and daily initialization features

Forty‐six aperture‐based features were calculated using the Python modules Pydicom (version 2.4.4) and DVHA‐MLCA (version 0.2.3. post1) (Table 1). Additionally, twenty‐one mechanical features were defined for each control point,^40^, ^43^, ^47^ including leaf positions, jaw positions, leaf velocity and acceleration, leaf movement direction, leaf movement status (starting, stopping, resting, or moving), leaf acceleration or deceleration status, leaf bank number, borders of each leaf (top, bottom, left, and right), collimator and gantry angle, gravity vector,^47^ monitor unit values, the number of control points per beam (field), and number of beams per plan.

**TABLE 1:** List of forty‐six aperture‐based features extracted from DICOM‐RT files.

Feature	Level^2^	Brief description
Leaf Sequence Variability (LSV)^49^	CP^1^	Variability of adjacent leaf positions relative to the maximum leaf position within each bank
Aperture Area Variability (AAV)^49^	CP	Aperture area at a control point relative to the maximum aperture area across the beam.
Modulation Complexity Score (MCS_cp_)^49^	CP	Product of LSV and AAV
Modulation Complexity Score (MCS_beam_)^49^	Beam	Monitor Unit (MU) weighted product of LSV and AAV
Leaf Perimeter‐to‐Aperture Area Ratio	CP/Beam	Ratio of the combined leaf‐end and leaf‐side perimeters to the corresponding aperture area
Aperture Area (AA)^50^	CP	Area enclosed by the MLC‐defined aperture
Aperture Perimeter (AP)^50^	CP	Perimeter of the MLC‐defined aperture
Beam area/perimeter (BA/ BP)^50^	Beam	MU‐weighted of AA and AP across the beam
Aperture Irregularity (AI_cp_)^50^	CP	Square of the aperture perimeter divided by 4π times of the aperture area
Aperture Irregularity (AI_beam_)^50^	Beam	MU‐weighted product of AI_cp_
Beam Modulation (BM)^50^	Beam	MU‐weighted aperture area relative to the union of apertures across the beam.
Younge complexity score (Edge Metric)^51^	CP	Ratio of the weighted leaf‐end and leaf‐side perimeters to the corresponding aperture area.
Complete Irradiated Area Outline (CIAO) perimeter^28^	Beam	Perimeter of the region defined by the maximum opening of each leaf across the beam.
CIAO area^28^, ^52^	Beam	Area enclosed by the complete irradiated area outline.
CIAO Area‐to‐Perimeter Ratio^28^	Beam	Ratio of CIAO area to CIAO perimeter.
CIAO irregularity factor^28^	Beam	Irregularity measure of the complete irradiated area outline.
Unique Opening Index (UOI)^52^	CP	Quantifies the number of open leaves within the aperture.
Leaf Travel Index (LTI)^53^	Beam	Total distance traveled by each leaf within a beam
LTI modulation complexity score ^53^	CP	Product of LTI and MCS.
Cross Axis Score (CAS)^54^	CP	Fraction of open leaves crossing the beam central axis.
Closed‐Leaf Score (CLS)^54^	CP	Fraction of closed leaves within the jaw‐defined aperture.
Small Aperture Score (SAS)^54^	CP	Fraction of leaf‐pair gaps below 2, 5, 10, or 20 mm.
Maximum leaf pair gap^28^	Beam	Quantifies maximum leaf‐pair gaps across all control points of a beam
Leaf‐Pair Gap Categories	CP	Classification of leaf‐pair gaps as < 2, < 5, < 10, or < 20 mm.
Jaw area,^28^	CP	Area enclosed by the treatment jaws.
Aperture‐to‐Jaw Area Ratio^28^	CP	Ratio of aperture area to jaw‐defined area.
Leaf pair gap^28^	CP	Distance between the opposing leaves of each leaf pair
Aperture‐to‐Circle Area Ratio^28^	CP	Ratio of aperture area to the area of a circle with radius 50, 100, 150, or 200 mm.
Mean Aperture Displacement (MAD_cp_).^55^	CP	Mean distance between the leaf‐pair midline and the beam central axis.
Mean Aperture Displacement (MAD_beam_).^55^	Beam	MU‐weighted product of MAD_cp_
Fraction of Small Leaf‐Pair Gaps^28^	Beam	Fraction of control points with leaf‐pair gaps below 2, 5, 10, or 20 mm.
Leaf‐Pair Gap Distribution Statistics^28^	Beam	Mean, variance, skewness, and kurtosis of the leaf‐pair gap distribution across the beam.

^1^CP: control point.

^2^The “Level” column indicates whether the parameter was calculated at the control‐point or beam level.

In addition, eleven daily machine‐specific parameters for each MLC leaf were incorporated into the machine learning model. Before patient treatment, the Linac undergoes an initialization procedure that records several parameters in “.init” files, which must remain within the predefined tolerances to enable treatment. These parameters included the backlash, the degree of leaf spring compression, the minimum and maximum raw secondary readouts of each leaf potentiometer, the maximum error during the secondary calibration of each leaf, the average Pulse Width Modulation (PWM) during the touch test, the leaf pair gap values during the touch test, and the daily, the monthly, the annual, and the lifetime traveled distance of each leaf (leaf odometer).^48^ All parameters were extracted using a self‐written Python script (version 3.10). A Spearman correlation coefficient was employed to assess the correlation between these features and the signed positional discrepancy of individual MLC leaves.

### 2.3 Calculation of leaf positional errors between RT‐Plan and Dynalog files

During treatment delivery with C‐series linear accelerators, the leaf position data are recorded in Dynalog files at 50 ms intervals, resulting in multiple temporal snapshots for each leaf. In contrast, the corresponding planned leaf positions are available at discrete control points within the DICOM‐RT files. Consequently, the number of recorded leaf positions in the Dynalog files exceeds the number of control points defined in the treatment plan. To accurately quantify leaf positional discrepancies, interpolation between the planned and delivered positions is necessary. The delivered leaf positions were analyzed using the log analyzer module of Pylinac (version 3.19). Additionally, cubic interpolation was implemented using an in‐house Python script (version 3.10) to align Dynalog snapshots with the control point data and compute the signed MLC positional errors.

### 2.4 Model construction and evaluation

To ensure complete independence between model development and evaluation, the dataset was partitioned at the treatment‐plan level, with each plan corresponding to a single patient. Importantly, for each patient, the Dynalog file and MLC initialization measurements associated with the corresponding treatment plan were obtained from only one treatment fraction. All MLC initialization parameters remained within the manufacturer‐defined tolerance limits. Approximately 80% of the plans (23 plans/patients) were allocated to the training/validation cohort, while the remaining 20% (6 plans/patients) were reserved as an independent test cohort. The test cohort comprised one prostate, three brain, and two head and neck plans/patients, whereas the training/validation cohort comprised two prostate, six brain, and fifteen head and neck plans/patients.

Seventy‐eight features (Section 2.3) and the positional discrepancies between the planned and delivered leaf positions were extracted for each leaf at every control point, defining a single sample. This yielded a two‐dimensional feature matrix of shape (number of samples × number of features) and a corresponding target vector of leaf positional errors with shape (number of samples,). The resulting training/validation dataset comprised 2,322,000 samples, while the independent test dataset contained 445,680 samples.

Owing to the continuous nature of the data, the LightGBM algorithm, as an advanced machine learning model, was employed to produce more generalized outcomes. LightGBM, a tree‐based algorithm, discretizes continuous features using histogram‐based methods, thereby reducing memory usage and speeding up computations. This efficiency enables LightGBM to handle large‐scale datasets efficiently while yielding a more accurate and generalized model.^56^

The feature matrix served as model input, and the signed leaf positional discrepancies were used as ground‐truth targets for regression. To prevent data leakage during model development, treatment‐plan identifiers were retained during data preparation, and a treatment‐plan‐grouped three‐fold cross‐validation (GroupKFold) strategy was used. Thus, all samples originating from the same treatment plan were assigned exclusively to a single fold, and no information from a given treatment plan was shared between training and validation folds.

Hyperparameter optimization was performed on the training/validation cohort using a sequential grid‐search strategy. Predefined values were evaluated for each hyperparameter using three‐fold GroupKFold cross‐validation. This cross‐validation was used for hyperparameter selection rather than as an inner component of a nested cross‐validation framework. For each parameter value, the model was trained on two folds and evaluated on the remaining fold, and the mean validation Root Mean Square Error (RMSE) across the three folds was calculated. The value yielding the lowest mean validation RMSE was selected, fixed, and used in the subsequent optimization step. The values considered for each hyperparameter are provided in Table S1.

The key hyperparameters tuned included: boosting type (e.g., gradient boosting decision tree (GBDT), random forest (RF), etc.), objective (the loss function used during training), the number of boosting iterations or trees, the maximum depth of each tree, the maximum number of leaves per tree, learning rate (the shrinkage rate per iteration), random seed (for reproducible results), data sampling strategy (e.g., bagging or Gradient‐based One‐Side Sampling (GOSS)), the subset of features used in each iteration/tree, bagging fraction (the proportion of data selected in each tree), the frequency of bagging, the minimum data in each leaf, and L1/L2 regularization parameters.^56^

After hyperparameter optimization, a second three‐fold GroupKFold cross‐validation was performed on the same training/validation cohort using the final fixed hyperparameter configuration. Mean Absolute Error (MAE) and RMSE were calculated across the three folds to characterize the performance of the optimized configuration. The optimized configuration was subsequently used to retrain the model on the entire training/validation cohort. Feature selection was then performed using the gain‐based feature‐importance scores obtained from this model.^56^ Features accounting for 98% of the cumulative feature importance were retained, reducing the feature set from 78 to 25 features. The final model was subsequently retrained from scratch on the entire training/validation cohort using the selected features and the optimized hyperparameter configuration. The resulting model was saved and evaluated on the independent held‐out test cohort, which was not used during hyperparameter optimization or feature selection. Final model performance on the test cohort was assessed using MAE, RMSE, and R^2^ score.

All computations were executed on an ASUS X415 system with an 11th Gen Intel® Core™ i5‐1135G7 processor running at 2.42 GHz. The total computation time was approximately 2 minutes.

### 2.5 DVH analysis and model predictability for different treatment fractions

Following model training, validation, and testing, an independent dataset comprising 9 additional IMRT plans from 9 unique patients (3 prostate, 2 brain, and 4 head and neck cases) was used for the clinical dosimetric evaluation. These 9 plans were completely separate from the 29 IMRT plans used for model development and were entirely excluded from the training, validation, and testing processes. The corresponding Dynalog files were collected over six consecutive treatment fractions for each plan, with the initialization and log files acquired on different treatment days from those used for model development.

Input features were extracted and the signed leaf positional errors were calculated for these clinical data. Then, the features fed into the model and the predicted discrepancies were acquired separately for the six treatment fractions. The dosimetric effects of these errors were analyzed for various treatment sites, with a specific focus on the first treatment fraction. To perform this analysis, the predicted leaf positions (calculated as the plan position plus the predicted error) and the delivered leaf positions, were integrated separately into the primary DICOM‐RT files. Subsequently, dose calculations for the predicted, delivered, and planned leaf positions were performed using Eclipse TPS on the selected patients' computed tomography (CT) images and RT structures, with a 2.5 mm dose calculation grid and the AAA dose calculation algorithm. From these calculations, dosimetric parameters were obtained from DVHs.

The dosimetric analysis focused on the extraction of DVH parameters for the Gross Tumor Volume (GTV), Clinical Target Volume (CTV), and Planning Target Volume (PTV) as targets, as well as for Organs at Risk (OARs). The considered parameters for the targets were D_98%_, D_95%_, D_2%_ (the doses received by 98%, 95%, and 2% of the volume, respectively), D_mean_ (mean dose). Volume‐based metrics included V_107%_, V_100%_, and V_95%_, corresponding to the percentages of the target volume receiving at least 107%, 100%, and 95% of the prescribed dose, respectively. In addition, the Homogeneity Index (HI)^57^ and Conformity Index (CI)^58^ were calculated to assess dose uniformity within the target and the degree of conformity of the prescribed dose distribution to the target volume, respectively.

For OARs, D_max_ (maximum dose), D_mean_, V_10%_, V_25%_, and V_50%_ (the volumes receiving 10%, 25%, and 50% of the prescribed dose, respectively) were obtained.

Moreover, the precision of the model in predicting the signed daily positional errors was evaluated across different treatment fractions for various treatment sites.

### 2.6 GPR analysis

To evaluate the agreement among the planned dose distribution and the dose distributions calculated using the delivered and predicted MLC leaf positions, GPRs were calculated for nine independent IMRT plans. Three pairwise comparisons were performed: planned versus delivered, planned versus predicted, and delivered versus predicted, with the latter providing a direct assessment of the agreement between the predicted and delivered dose distributions.

The dose distributions were calculated using a 2.5‐mm dose grid. To provide finer spatial sampling for the gamma analysis, the dose distributions were interpolated to a common 1.0‐mm grid using three‐dimensional linear interpolation. Gamma analysis was subsequently performed independently on each axial slice using Pylinac (version 3.19) and represents a 2D, slice‐by‐slice gamma evaluation. Global dose normalization was used, and dose points receiving less than 10% of the maximum reference dose were excluded from the analysis. Gamma analysis was performed using the criteria of 3%/3 mm, 3%/2 mm, 2%/2 mm, and 1%/1 mm, with a passing criterion of 95%.

For comparison with conventional measurement‐based PSQA, previously available GPR results from Delta4 phantom measurements (Delta4 Phantom+, ScandiDos AB, Uppsala, Sweden) were included for the nine IMRT plans. The available measurement‐based GPRs were obtained using a 3%/3 mm gamma criterion, a 10% dose threshold, and a 95% passing criterion.

## 3 RESULTS

### 3.1 Optimized model performance

During the hyperparameter optimization procedure, the optimal hyperparameter values for the LightGBM algorithm included a boosting type of GBDT, with a Mean Squared Error (MSE) loss function (L2 regression) as the objective, a maximum depth of ‐1, 51 leaves, 500 boosting iterations, a learning rate of 0.050, a random seed of 42, and a data sampling strategy of bagging. The feature and bagging fractions were both 1.000, with a bagging frequency of 0.000. Additionally, the best results were achieved with a minimum of 120 data per leaf, and L1 and L2 regularization strengths of 0.100 and 1.030, respectively (lambda_l1 = 0.100, lambda_l2 = 1.030). The evolution of the training and validation loss functions using these optimized hyperparameters during three‐fold cross‐validation is shown in Figure 1. Because MSE was used as the objective function, the learning curves represent the MSE as a function of the number of boosting iterations. Both training and validation losses decreased progressively with increasing numbers of boosting iterations, indicating stable model convergence. The training loss decreased more rapidly and reached a lower value than the validation loss, while the validation loss decreased at a slower rate and remained consistently higher than the training loss. At 500 iterations, the final training and validation MSE values were approximately 0.006 and 0.027 mm^2^, respectively. Although the increasing separation between the training and validation curves indicates a modest degree of overfitting, the validation loss continued to decrease throughout the investigated range and did not show a clear increase or divergence. Therefore, extending the number of boosting iterations beyond 500 was not considered necessary, particularly because the additional improvement in validation loss became progressively smaller at higher iteration numbers.

**FIGURE 1:**
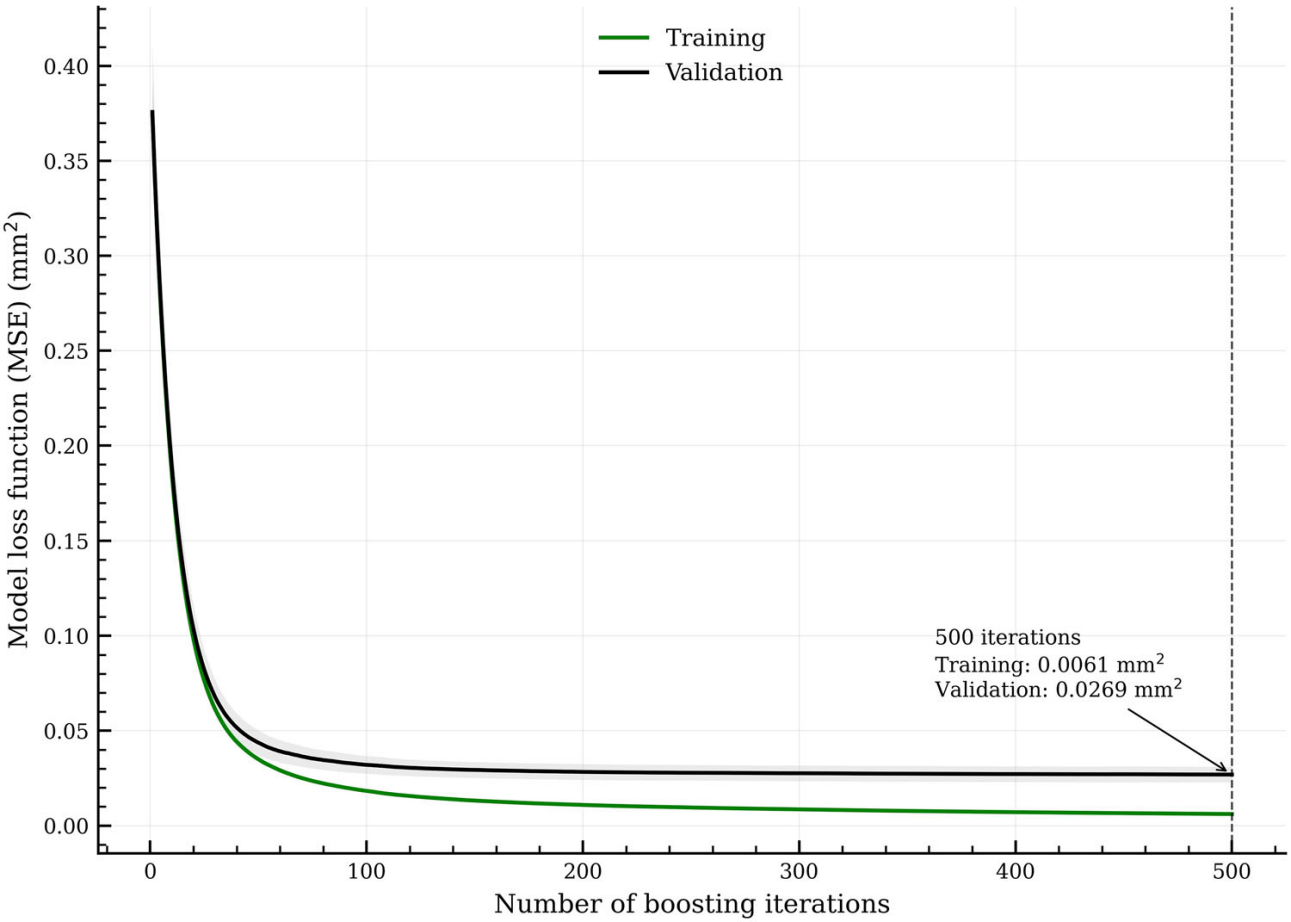
Learning curves of the LightGBM model with the optimized hyperparameters during three‐fold patient/plan‐level cross‐validation using 78 features, showing MSE for the training and validation datasets as a function of the number of boosting iterations. Shaded regions represent ± 1 standard deviation across the three cross‐validation folds. The vertical dashed line indicates the selected 500 boosting iterations.

The mean and standard deviation of the performance metrics across the three cross‐validation folds at the selected model configuration were 0.035 ± 0.001 mm for MAE and 0.078 ± 0.003 mm for RMSE in the training subsets, compared with 0.070 ± 0.005 mm for MAE and 0.164 ± 0.013 mm for the validation subsets. The higher validation errors compared with the corresponding training errors are consistent with the small generalization gap observed in the learning curves. Importantly, the cross‐validation results were obtained using the complete set of 78 features and were used primarily for hyperparameter optimization and assessment of model behavior across patient/plan‐level folds. Following cross‐validation and feature selection, the optimized model achieved an MAE of 0.054 mm and an RMSE of 0.144 mm on the independent test dataset. When evaluated on a per‐plan/patient basis, the model achieved a mean MAE of 0.046 mm and a mean RMSE of 0.125 mm. Stratified by treatment site, the lowest prediction errors were observed for the prostate plan, with an MAE of 0.025 mm and an RMSE of 0.083 mm. For the brain plans, the mean MAE and RMSE were 0.041 mm and 0.120 mm, respectively, whereas the head and neck plans showed higher mean errors, with an MAE of 0.064 mm and an RMSE of 0.153 mm.

Although these values were higher than the cross‐validation training performance, this difference is expected because the test dataset consisted of previously unseen patient/plan data. The independent test results provide the more relevant measure of the final model's generalization capability, while the learning curves demonstrate stable convergence during model development.

Figure 2(a) presents a strong correlation between the predicted and actual target values of the test dataset, achieving an R^2^ score of 0.932. Figure 2(b) demonstrates Bland‐Altman analysis of this data with strong agreement between the predicted and delivered leaf positional errors. The mean bias between predictions and ground‐truth values is approximately 0.003 mm, indicating the absence of systematic overestimation or underestimation by the LightGBM model. The 95% limits of agreement are −0.280 mm to 0.285 mm, showing that most prediction errors remain within a relatively narrow interval. Furthermore, the majority of differences cluster around zero, reflecting high predictive consistency for the clinically prevalent small MLC deviations. Although several outliers are observed at larger positional errors, no directional trend toward consistent positive or negative deviation is identified. These findings support the capability of the proposed model to accurately estimate daily MLC positional inaccuracies.

**FIGURE 2:**
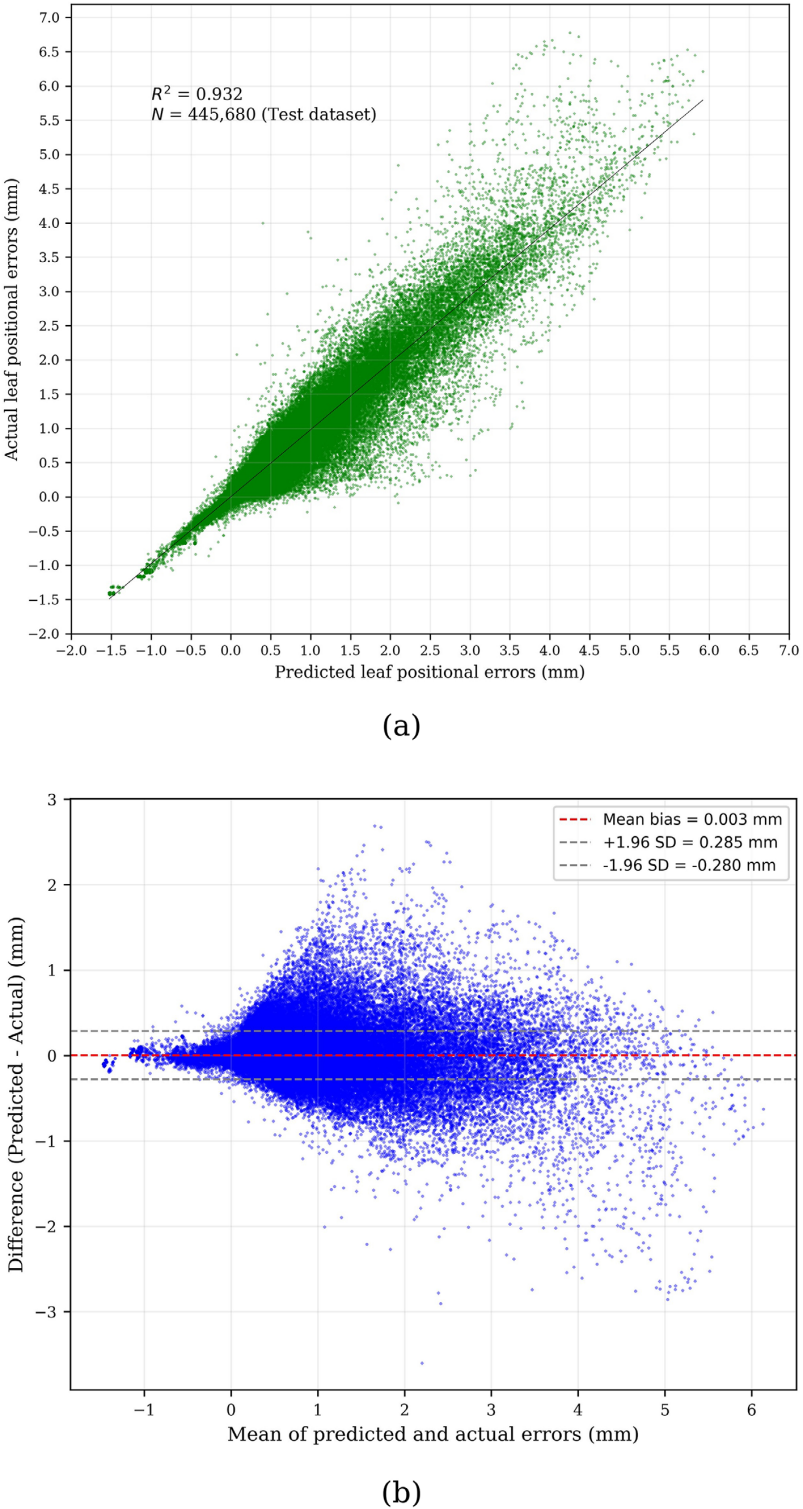
Model performance assessed using (a) correlation analysis and (b) Bland–Altman analysis between predicted and delivered leaf positional errors for test dataset. The dashed black line in (a) represents the line of best fit, with a coefficient of determination (R^2^) of 0.932. In (b), the dashed red line indicates the mean bias, and the dashed black lines denote the 95% limits of agreement.

Figure 3 illustrates the distribution of leaf positional errors across all control points of test dataset for each leaf. Specifically, Figure 3(a) depicts the differences between the planned and the delivered positions. The mean deviations are below 1 mm (–0.026 mm to 0.718 mm), with standard deviations ranging from 0.012 mm to 1.239 mm across all leaves. Planned positions generally exceed delivered positions, with larger discrepancies particularly evident in the central leaves. Figure 3(b) presents the predicted versus delivered errors. The close alignment between the predicted and delivered positions is reflected by lower standard deviations (0.013 mm to 0.364 mm) and mean errors ranging from –0.052 mm to 0.027 mm. These results indicate that the model accurately captures the majority of positional deviations.

**FIGURE 3:**
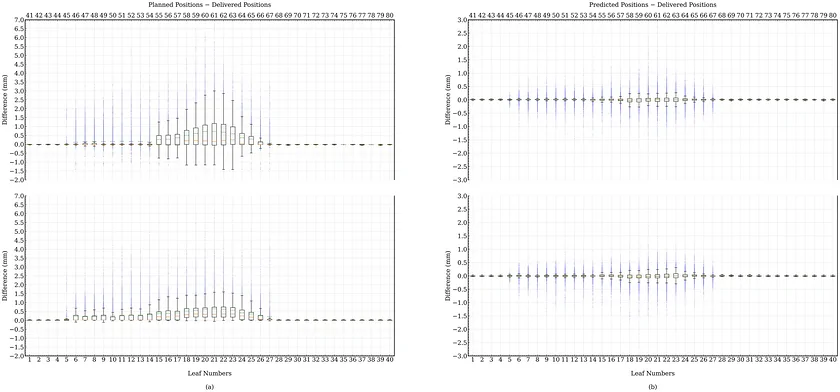
Differences between the planned and delivered (a), and predicted and delivered (b) leaf positions across all control points in the test dataset. For clarity, each subplot is divided into a bottom panel (leaves 1–40) and a top panel (leaves 41–80). Each box represents the distribution of positional errors for an individual leaf, with the orange and green horizontal lines indicating the median and mean values, respectively. The boxes extend from the first quartile (Q1) to the third quartile (Q3), while the whiskers extend to Q1 − 1.5 × IQR and Q3 + 1.5 × IQR, where IQR denotes the interquartile range. Data points falling outside this range represent larger positional errors and are shown as blue dots.

According to these results, the 90th, 95th, and 99th percentiles (P90, P95, and P99) of the absolute leaf positional errors during delivery relative to the planned positions were 0.68, 1.23, and 2.81 mm, respectively, with a maximum error of 6.78 mm. The maximum error occurred at only one control point, indicating that such large deviations were rare in the test dataset, whereas the majority of observations corresponded to relatively small positional errors. Despite their low frequency, the model reproduced the upper tail of the error distribution reasonably well. These relatively large errors, including observations exceeding the whisker limits, are shown as blue dots in Figure 3(a). The corresponding predicted values for the 90th, 95th, and 99th percentiles and the maximum error were 0.70, 1.26, 2.76, and 5.92 mm, respectively. The greater discrepancy between the delivered and predicted values at the upper end of the error distribution, including the larger deviations represented by the blue dots in Figure 3(b), may be attributed to the substantial predominance of small positional errors in the dataset. This imbalance results in comparatively fewer samples representing rare, large positional deviations, potentially limiting the model's ability to accurately learn and reproduce extreme errors.

The 25 features accounting for 98% of the cumulative feature importance during model training are presented in Table S2. Feature importance analysis identified leaf velocity, mean aperture displacement (MAD) at each control point, and the maximum raw secondary readout of each leaf potentiometer as the key predictors across the three feature categories. Spearman correlation analysis revealed that leaf velocity was the most influential feature (*ρ =* 0.703, *p*‐value < 0.001), highlighting its pivotal role in predicting positional errors. The leaf movement direction (i.e., motion toward or away from the midline) ranked as the second most important feature (*ρ* = ‐0.638, *p*‐value < 0.001), whereas MAD demonstrated moderate importance (*ρ* = –0.359, *p*‐value < 0.001). Additionally, the maximum raw secondary readout was found to be a relevant quality assurance parameter (*ρ* = 0.431, *p* < 0.001), along with the minimum raw secondary readout (*ρ* = 0.450, *p *< 0.001).

### 3.2 Model performance in daily error prediction and analysis of dosimetric indices

The DVH curves obtained from six consecutive treatment fractions for the three treatment sites (prostate, brain, and head and neck) are presented in Figure S1, while Figure 4 presents the DVHs from the first treatment fraction for representative cases from each treatment site. Structures exhibiting relatively large dosimetric deviations are shown. The six‐fraction analysis was performed to evaluate the consistency of the predicted daily MLC positional deviations and their corresponding dosimetric effects across repeated treatment fractions. As shown in Figure 4, the DVH curves calculated using the predicted MLC positions closely followed those obtained using the delivered MLC positions, while both exhibited deviations from the planned dose distribution. For example, in the brain case, the D_98%_ of the PTV decreased by 0.416 Gy for the delivered distribution and 0.364 Gy for the predicted distribution relative to the planned dose. The right optic nerve exhibited increases of 0.545 Gy and 0.533 Gy for the delivered and predicted distributions, respectively. In the prostate case, the corresponding deviations in PTV prostate were −0.228 Gy and −0.188 Gy. For the head and neck case, the deviations were −0.251 Gy and −0.114 Gy for PTV66 Gy, −0.289 Gy and −0.168 Gy for PTV60 Gy, and 0.374 Gy and 0.474 Gy for PTV54 Gy. Among the OARs, the right parotid gland exhibited the largest change, with increases in D_mean_ of 1.083 Gy and 1.103 Gy for the delivered and predicted distributions, respectively. The deviations of DVH curves corresponding to the delivered and predicted leaf positions in comparison with planned DVHs, are more apparent in the regions with steep fall‐off of the DVHs (Endpoint region). Overall, the DVH changes obtained from the predicted leaf positions closely reproduced the corresponding changes observed for the delivered leaf positions across the evaluated treatment fractions.

**FIGURE 4:**
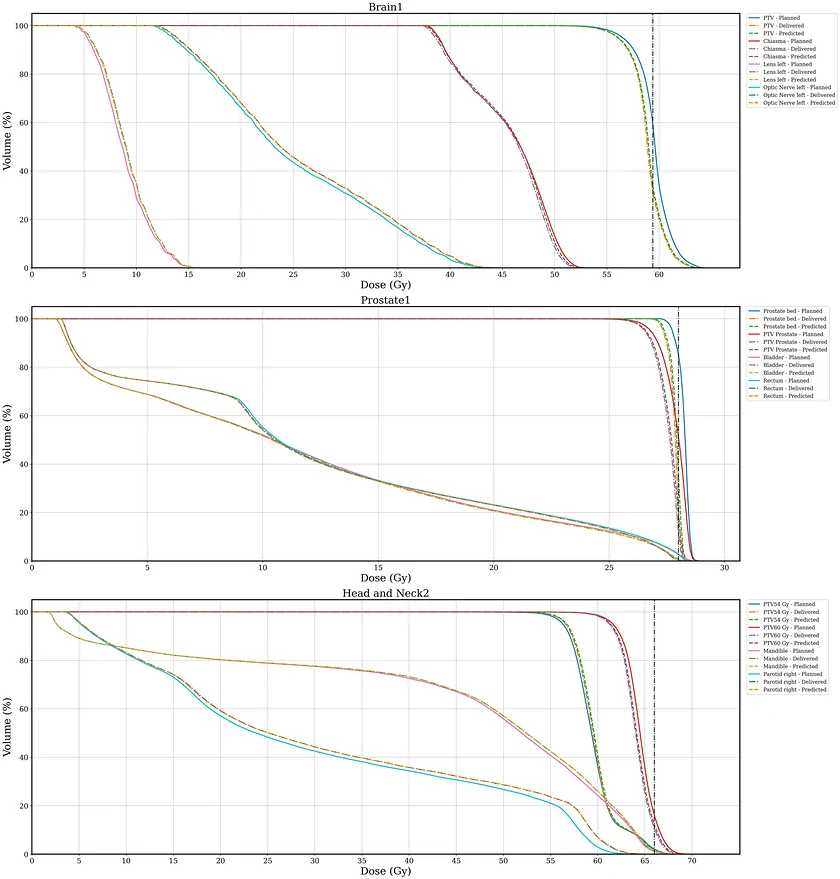
DVH curves derived from dose distributions corresponding to the planned, delivered, and predicted MLC positions of first treatment fraction and selected OARs and PTVs for three treatment sites: brain, prostate, and head and neck. Solid lines represent the planned, dashed lines correspond to the delivered, and dash‐dotted lines indicate the predicted. The vertical black dashed line indicates the prescribed dose for each case.

The mean, standard deviation, minimum, and maximum differences between the dosimetric indices obtained using the delivered and predicted leaf positions and those obtained from the planned positions for the nine IMRT plans during the first treatment fraction are summarized in Table 2. For the target structures, the mean deviations in D_98%_, D_95%_, D_2%_, and D_mean_ relative to the planned dose distribution ranged from −0.467 ± 0.355 Gy to −0.282 ± 0.372 Gy for the delivered leaf positions and from −0.346 ± 0.356 Gy to −0.233 ± 0.365 Gy for the predicted leaf positions, indicating comparable dosimetric deviations from the planned distribution.

**TABLE 2:** Dosimetric indices statistics for delivered‐planned and predicted‐planned dose comparisons in nine IMRT plans during the first treatment fraction.

Dosimetric indices	Targets (minimum, maximum, mean ± standard deviation)		OARs (minimum, maximum, mean ± standard deviation)	
	Delivered vs planned	Predicted vs planned	Delivered vs planned	Predicted vs planned
D_98%_ (Gy)	(‐1.056, 0.749, ‐0.282 ± 0.372)	(‐0.843, 0.617, ‐0.233 ± 0.365)	–	–
D_95%_ (Gy)	(‐1.051, 0.735, ‐0.292 ± 0.377)	(‐0.823, 0.590, ‐0.245 ± 0.370)	–	–
D_2%_ (Gy)	(‐0.986, 0.618, ‐0.467 ± 0.355)	(‐0.840, 0.654, ‐0.346 ± 0.356)	–	–
V_107%_ (%)	(‐1.128, 0.001, ‐0.112 ± 0.295)	(‐1.109, 0.001, ‐0.096 ± 0.255)		
V_100%_ (%)	(‐83.030, 0.001, ‐10.314 ± 16.341)	(‐64.625, 0.001, ‐8.819 ± 13.284)	–	–
V_95%_ (%)	(‐13.846, 0.771, ‐2.084 ± 3.106)	(‐18.814, 1.108, ‐2.074 ± 3.828)	–	‐
HI	(‐0.234, 0.007, ‐0.008 ± 0.032)	(‐0.192, 0.009, ‐0.005 ± 0.026)	–	–
CI	(‐0.349, 0.000, ‐0.085 ± 0.090)	(‐0.353, 0.000, ‐0.074 ± 0.087)	–	–
D_mean_ (Gy)	(‐1.031, 0.819, ‐0.350 ± 0.383)	(‐0.804, 0.795, ‐0.274 ± 0.378)	(‐0.655, 1.103, 0.046 ± 0.237)	(‐0.760, 1.083, 0.062 ± 0.248)
D_max_ (Gy)	–	–	(‐0.717, 2.424, ‐0.052 ± 0.478)	(‐0.767, 2.603, 0.028 ± 0.478)
V_50%_ (%)	–	–	(‐0.927, 3.027, 0.175 ± 0.683)	(‐1.753, 3.676, 0.166 ± 0.768)
V_25%_ (%)	–	–	(‐1.404, 2.116, 0.106 ± 0.539)	(‐0.813, 2.147, 0.118 ± 0.493)
V_10%_ (%)	–	–	(‐1.447, 2.967, 0.026 ± 0.450)	(‐0.952, 2.571, 0.039 ± 0.387)

The volumetric indices V_107%_, V_100%_, and V_95%_ showed comparable deviations from the planned values for the dose distributions obtained from the delivered and predicted MLC leaf positions. Across these three indices, the mean deviations ranged from −10.314 ± 16.341% to −0.112 ± 0.295% for the delivered leaf positions and from −8.819 ± 13.284% to −0.096 ± 0.255% for the predicted leaf positions. For the OARs, the deviations were generally smaller. The Dmax deviations from the planned values were −0.052 ± 0.478 Gy and 0.028 ± 0.478 Gy for the dose distributions obtained from the delivered and predicted MLC leaf positions, respectively, while the corresponding D_mean_ deviations were 0.046 ± 0.237 Gy and 0.062 ± 0.248 Gy. The volumetric indices V_50%_, V_25%_, and V_10%_ showed somewhat greater variability but exhibited similar trends between the dose distributions obtained from the delivered and predicted MLC leaf positions.

Although the mean deviations of the target dose indices were relatively small, the observed minimum and maximum deviations of approximately ± 1 Gy indicate that larger plan‐specific changes can occur. In particular, V_100%_ showed substantial variability, with the minimum deviation approaching −83%. For OARs, maximum deviations of approximately 3 Gy in D_max_ and 1 Gy in D_mean_ were observed in the dose distributions obtained from both the delivered and predicted MLC leaf positions. Similarly, the maximum deviations in V_50%_, V_25%_, and V_10%_ were 3.027%, 2.116%, and 2.967%, respectively, for the dose distributions obtained from the delivered leaf positions compared with the planned dose distribution, and 3.676%, 2.147%, and 2.571%, respectively, for those obtained from the predicted leaf positions. The conformity and homogeneity indices also demonstrated deviations from the planned values, with similar trends observed for the dose distributions obtained from the delivered and predicted leaf positions. These results indicate that the model was able to reproduce the clinically relevant dosimetric deviations associated with the delivered MLC positional errors during treatment delivery.

The distribution of dosimetric indices during the first treatment fraction is presented in Figure 5. Both delivered and predicted plans demonstrate closely aligned patterns of deviation from the planned reference, with narrow distributions for OARs and wider spreads for target indices. These findings indicate that the predictive model reliably reproduces the dosimetric deviations observed in treatment delivery. Notably, target volumes exhibit predominantly negative deviations across all dosimetric indices, whereas OARs show slight positive deviations. Overall, the magnitude of deviations is consistently smaller for OARs than for target structures.

**FIGURE 5:**
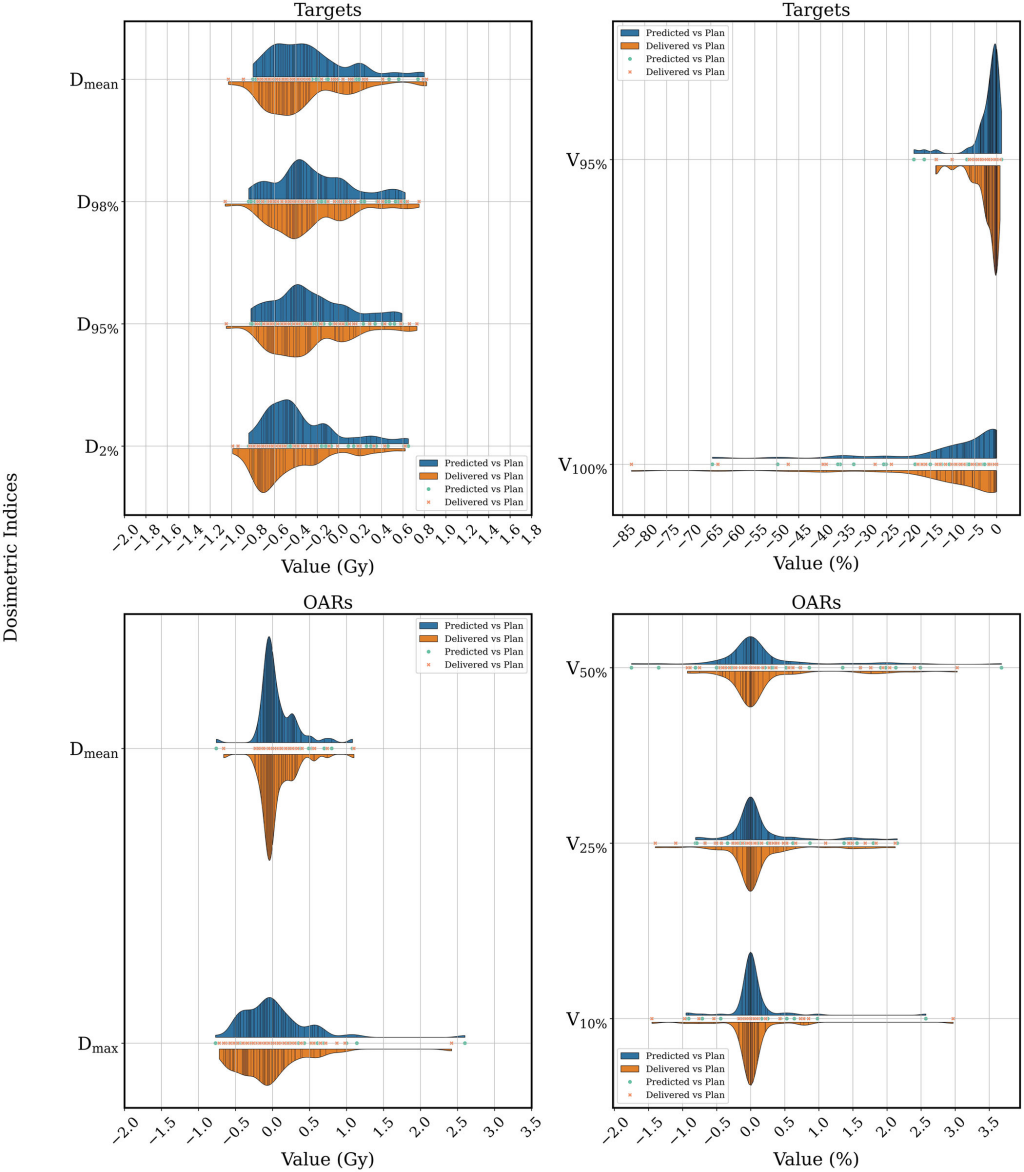
Distribution of dosimetric index deviations from the planned values for the dose distributions obtained from the delivered and predicted MLC leaf positions for target and OAR structures across nine IMRT plans for the first treatment fraction. The upper (blue) and lower (brown) lobes of the violin plots represent the deviations associated with the delivered and predicted MLC leaf positions, respectively. Dark vertical lines within each plot, along with scatter points (green circles and orange crosses) between the lobes, are included for enhanced visualization.

To further investigate the relationship between MLC positional deviations (delivered and predicted) and their dosimetric consequences, the 95th percentile of the absolute leaf positional error was calculated for each of the six consecutive treatment fractions, and the mean of these six values was then calculated for each selected treatment case (Figure 6). Subsequently, the effects of the positional deviations on dosimetric parameters and their correlations were evaluated separately for each clinical case (Figures 7 and 8). According to Figure 6, the head and neck cases generally exhibited larger positional deviations than the prostate and brain cases, which may be related to their greater treatment complexity and more extensive and dynamic MLC motion. In particular, the mean leaf velocity, averaged across all leaves within each plan, was higher for the head and neck cases than for the prostate and brain cases, consistent with the observed correlation between leaf velocity and positional errors. In addition, the proposed model was able to closely reproduce the delivered 95th percentile of the absolute leaf positional errors across all treatment cases.

**FIGURE 6:**
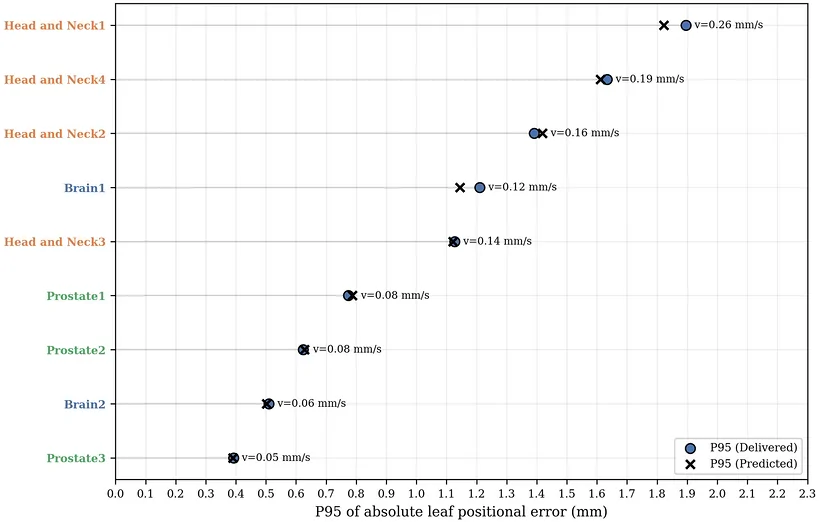
Comparison of the P95 of the delivered and predicted absolute leaf positional error averaged over six consecutive treatment days for 9 IMRT plans of different cases. Circles indicate the delivered values and crosses indicate the predicted values. The mean leaf velocity is shown for each treatment plan, and the colors of the case labels indicate the treatment site (head and neck, prostate, and brain).

**FIGURE 7:**
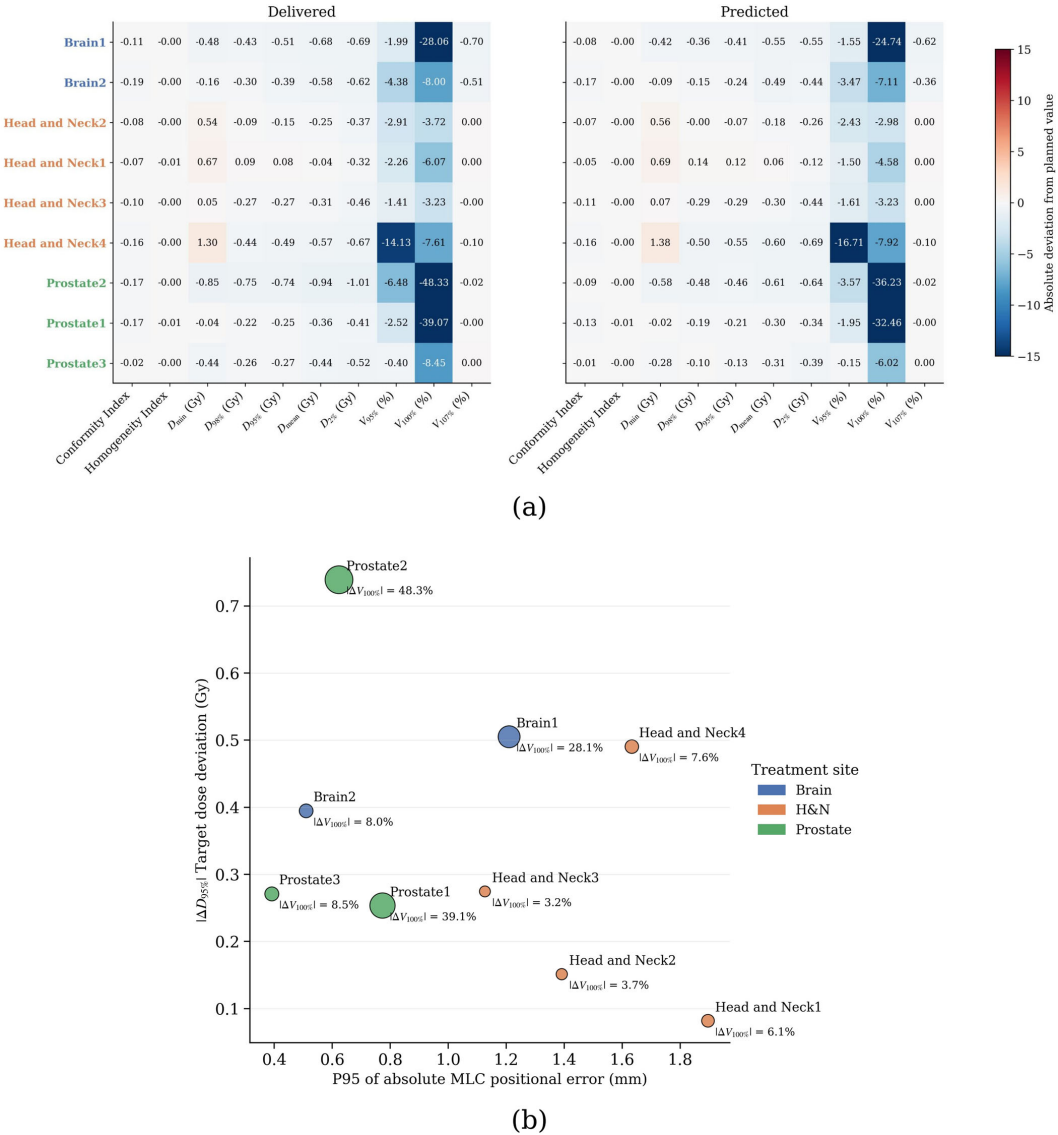
Dosimetric parameter differences averaged across treatment fractions extracted from dose distributions of delivered and predicted leaf positions relative to the plan (a), and correlations between the 95th percentile of the absolute MLC positional errors and the most deviated PTV dosimetric parameters, V_100%_ and D_95%_ of delivered dose distribution for each case relative to plan (b).

**FIGURE 8:**
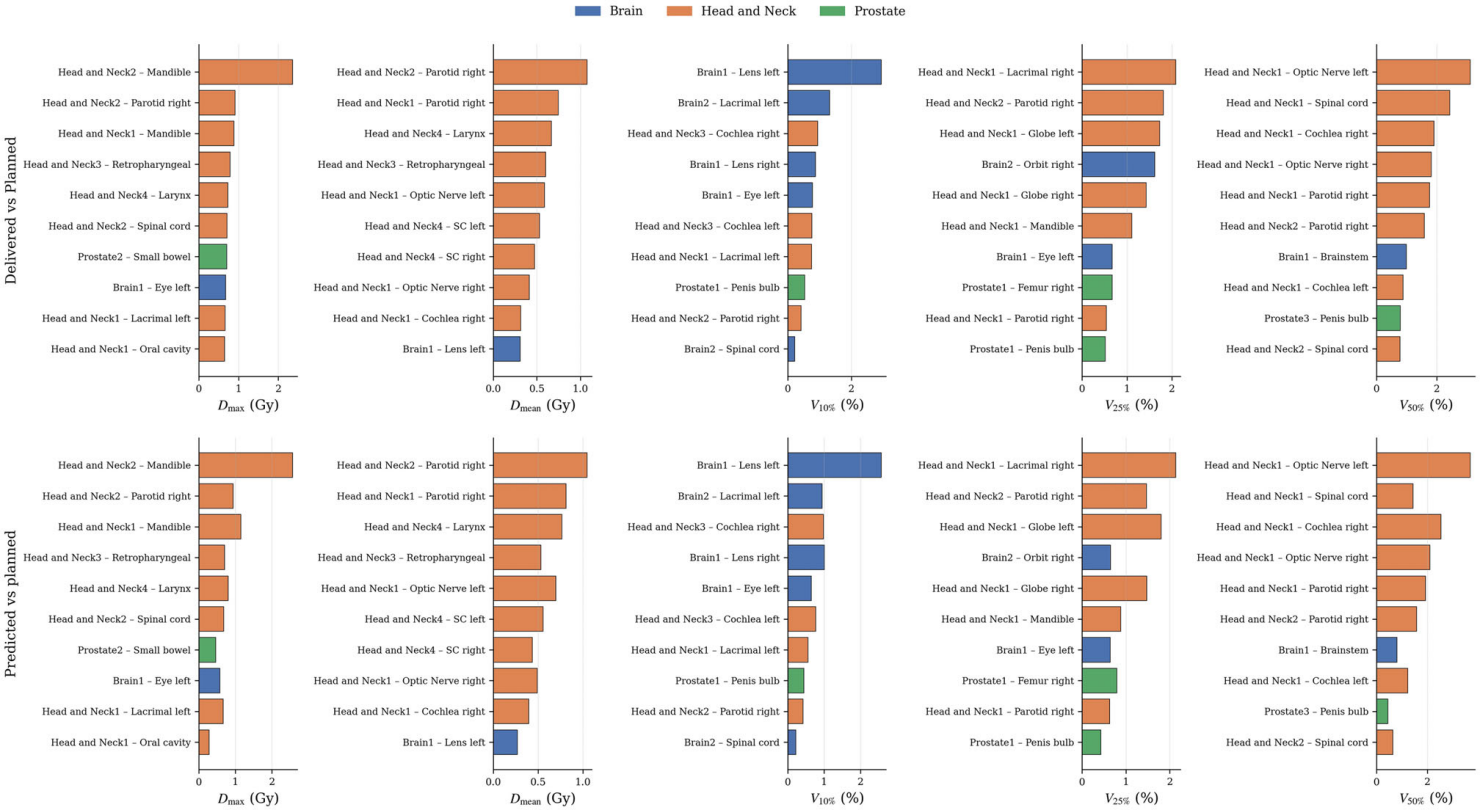
Top 10 OAR structures showing the largest absolute deviations in dosimetric parameters between the delivered and planned, and predicted and planned dose distributions, averaged over six consecutive treatment fractions across the nine IMRT cases.

Figure 7(a) presents the effects of delivered and predicted leaf positional deviations on the dosimetric indices of the PTVs for the evaluated cases, averaged across the treatment fractions. Among the evaluated indices, V_100%_ and D_95%_ exhibited the largest discrepancies. The correlations between the 95th percentile of the absolute delivered MLC positional errors and the corresponding deviations in these two dosimetric indices for the different treatment cases are presented in Figure 7(b). No consistent relationship was observed between the 95th percentile of MLC positional errors and the corresponding dosimetric deviations across the different treatment sites. This finding suggests that the dosimetric impact of MLC positional deviations cannot be adequately characterized by a single summary measure of positional error, such as the 95th percentile. The effects of MLC positional deviations on the dosimetric indices of OARs are illustrated in Figure 8 for the ten OARs showing the largest deviations across the evaluated cases. The predicted dosimetric parameters for each OAR structure are also presented in Figure 8 and showed close agreement with the corresponding values obtained from the delivered MLC positions. These findings highlight the cumulative effect of numerous individual MLC positional deviations occurring throughout treatment delivery on different regions of the target and OAR structures, resulting in varying changes in their respective dosimetric parameters.

To illustrate fraction‐to‐fraction variations in MLC leaf positional deviations, the maximum deviations of the delivered and predicted leaf positions from their corresponding planned positions for each leaf over six consecutive treatment fractions were evaluated for a head and neck case and are presented in Figure 9. A case exhibiting relatively large fraction‐to‐fraction fluctuations was selected for this analysis. The pattern of delivered leaf positional discrepancies relative to the planned positions for this plan across the six treatment fractions indicates that the day‐to‐day variations were generally small, although the maximum positional deviations from the planned positions reached approximately 6–7 mm for some leaves during each treatment fraction. A similar pattern was observed for the predicted positional deviations relative to the planned positions. Accordingly, their direct impact on the daily DVHs was relatively limited, as also demonstrated in Figure S1. Nevertheless, small inter‐fraction positional variations across multiple leaves can cumulatively produce measurable dosimetric changes during different treatment fractions relative to the plan. In the selected head and neck case, the cumulative effect of these variations resulted in a dosimetric deviation of up to approximately 1.5% in V_100%_.

**FIGURE 9:**
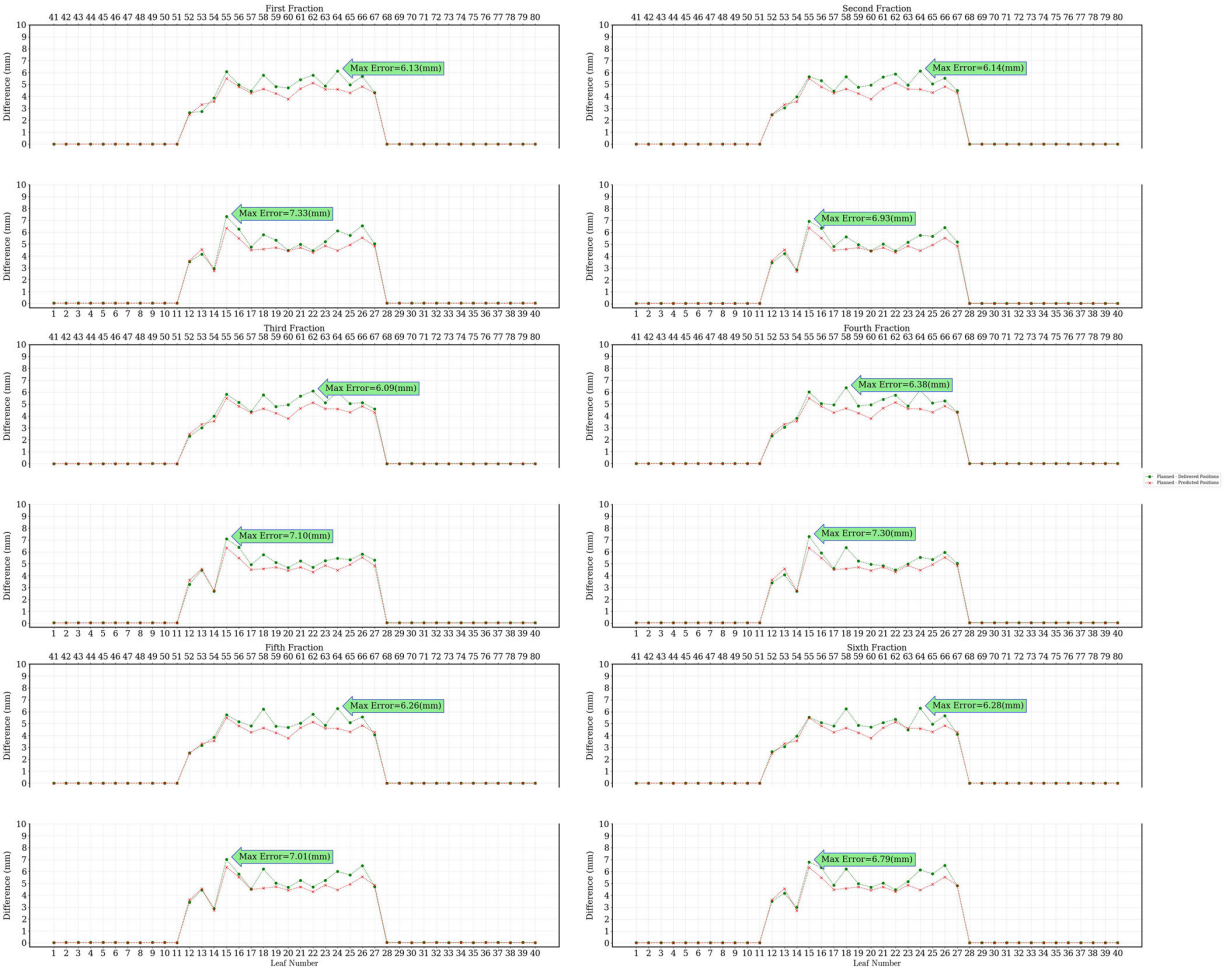
Comparison of the maximum delivered and predicted positional deviations of individual MLC leaves from their planned positions across six consecutive treatment fractions of a head and neck IMRT plan. The leaf exhibiting the maximum delivered positional deviation in each MLC bank is indicated by a green arrow. For clarity, leaves 1–40 are shown at the bottom panel, and the top panel shows leaves 41–80.

In this case, the delivered positional deviation of leaf 15 changed by 0.54 mm between the first and sixth fractions, decreasing from 7.33 mm to 6.79 mm. The variations in the maximum positional deviations of the delivered positions relative to the planned positions observed for leaves 58, 62, and 64 further illustrate that the leaf exhibiting the maximum positional deviation can vary across different treatment fractions. The proposed model was able to approximately reproduce the observed trends in these daily variations in the delivered positional deviations, with a maximum difference of less than 2 mm between the delivered and predicted curves. However, its ability to predict small fraction‐to‐fraction changes remained limited. The relatively limited predictive accuracy for these fraction‐to‐fraction changes may be related to the small magnitude of these inter‐fraction changes and to the relatively lower contribution of daily initialization features to the overall model prediction.

The changes of the initialization parameters over six consecutive treatment days are presented in Figure S2. The values from the first day were used as a reference, and the differences on subsequent days were calculated relative to this baseline for all leaves. The relative differences of each parameter were then averaged across the six days for each leaf within the MLC banks. As observed, the lifetime and annual odometer parameters remain essentially unchanged (Figure S2 (b)), indicating negligible variations over the evaluated period. In contrast, the monthly odometer represents a broad peak (Figure S2 (b)), reflecting increased movement of the central leaves. Other parameters show variable fluctuations, demonstrating their daily variability and potential impact on leaf positional errors.

In particular, the fluctuations in the maximum raw secondary readout are presented in Figure 10, both for individual leaves averaged across the treatment fractions relative to the first fraction as the reference and for individual treatment fractions averaged across the leaves. These results indicate that daily variations in this initialization parameter occur at both the leaf and fraction levels.

**FIGURE 10:**
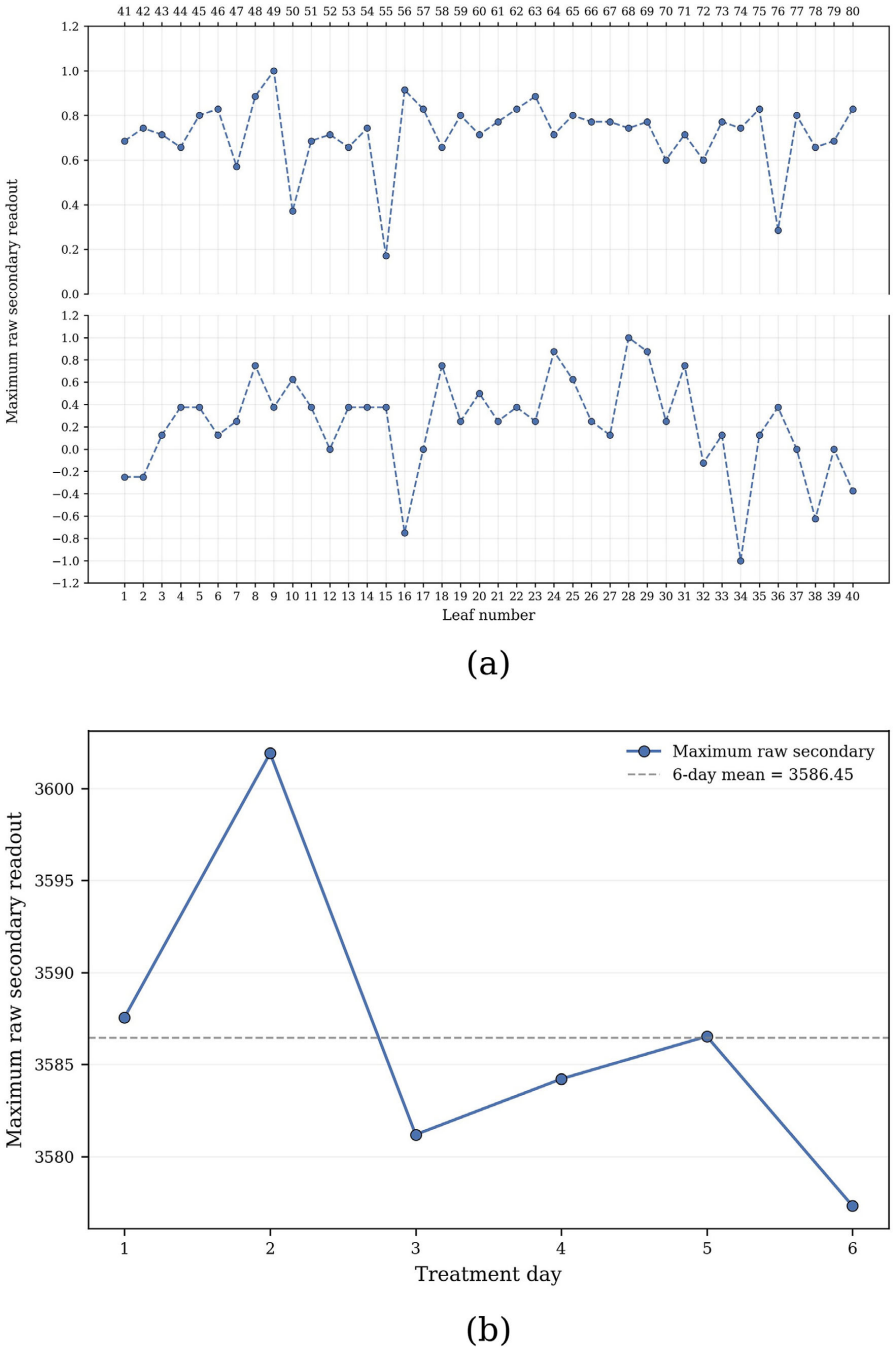
Variations of max raw secondary relative to the first treatment day, averaged across six consecutive treatment days for each leaf (a), variation of maximum raw secondary readout averaged across all leaves at each treatment day (b). The bottom panel shows leaves 1–40, and the top panel shows leaves 41–80.

To further demonstrate the potential cumulative effect of daily MLC variations, Figure 11 presents the daily V_100%_ for a prostate case that exhibited the largest daily dosimetric fluctuations among the evaluated cases. As shown in Figure 11, the delivered V_100%_ varied by approximately 4.4% across the treatment fractions. Although the model approximately reproduced the daily V_100%_ values, these findings suggest that in‐tolerance variations in daily MLC initialization measurements, particularly the maximum raw secondary readout, may provide useful information regarding fraction‐to‐fraction changes in MLC positional behavior.

**FIGURE 11:**
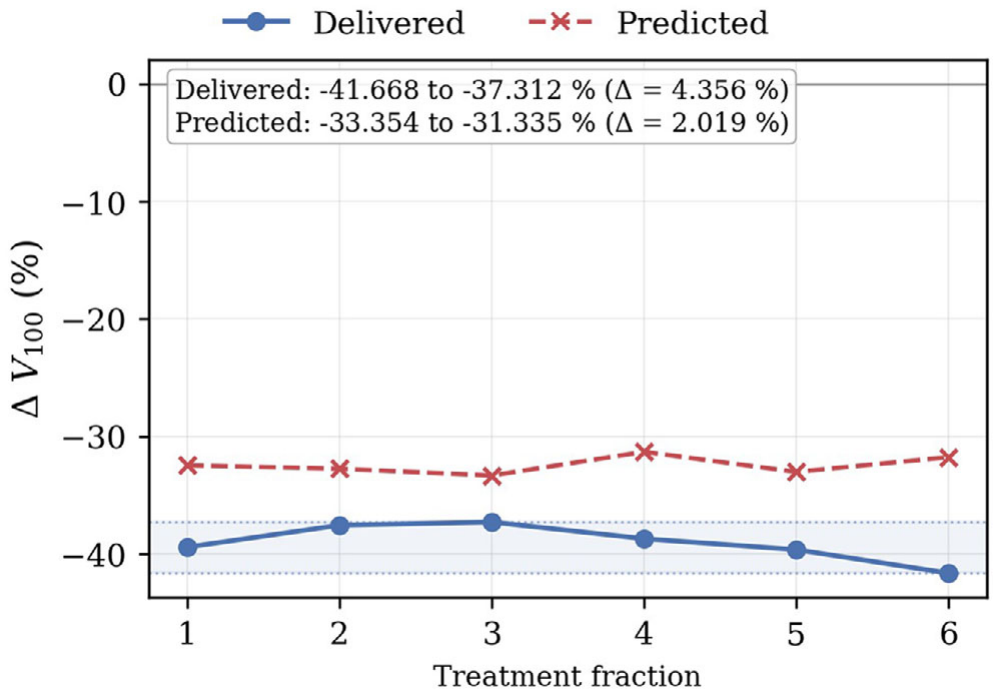
Variation in the V100% of PTV across six consecutive treatment days for a prostate patient with the largest observed deviation in this parameter. The difference between the delivered maximum and minimum V100% are represented in the light blue region.

### 3.3 GPR analysis

The evaluation of GPRs showed average values of 99.99 ± 0.01%, 99.98 ± 0.02%, 99.96 ± 0.14%, and 96.49 ± 3.56% for the 3%/3 mm, 3%/2 mm, 2%/2 mm, and 1%/1 mm criteria, respectively, for dose distributions of delivered leaf positions versus planned ones of 9 IMRT. Similarly, for the dose distribution acquired from predicted leaf positional errors versus planned ones, the corresponding GPRs were 99.99 ± 0.01%, 99.98 ± 0.02%, 99.93 ± 0.15%, and 97.01 ± 3.42%. In contrast, the previously acquired measurement‐based GPRs ranged from 97.00% to 100% under the 3%/3 mm criterion, which were generally lower than the corresponding log‐file‐based GPRs.

For the comparison between the dose distributions calculated using the delivered and predicted MLC positions, the corresponding GPRs were 99.99 ± 0.01%, 99.99 ± 0.01%, 99.99 ± 0.01%, and 97.01 ± 0.02% for the four criteria, respectively. These results demonstrate that the model consistently achieved comparable GPRs for the dose distributions calculated using the delivered and predicted MLC positional errors across the evaluated gamma criteria.

Although some differences were observed in the dosimetric indices of the delivered and predicted doses compared with the planned doses, the high GPR values indicate that all plans remained clinically acceptable for delivery.

## 4 DISCUSSION

In this study, we proposed using the LightGBM machine learning algorithm to predict daily MLC errors between planned and delivered positions at each control point of IMRT plans. The proposed approach was designed to address some challenges of conventional PSQA while complementing, rather than replacing it. By providing plan‐specific information on expected MLC positional deviations and their potential dosimetric consequences, it enables daily PSQA and plan‐specific evaluation of dose distributions and DVH parameters.

Given that, disagreement between delivered and calculated dose distribution is more strongly related to aperture‐based metrics in IMRT,^28^ we included these parameters to represent the deliverability of irregular apertures produced in IMRT plans alongside MLC initialization and mechanical parameters.

Among previous studies focused on predicting MLC leaf positional errors, the ANN model developed by Osman et al. achieved an MSE of 0.0001 mm^2^ and an R^2^ score of 0.9999 for leaf‐position prediction.^41^ The best‐performing model in the study by Lay et al. achieved an MAE of 0.004 mm for IMRT and 0.021 mm for VMAT. The robustness analysis demonstrated good agreement between the predicted and delivered values, with R^2^ scores of 0.966 for IMRT and 0.906 for VMAT.^43^ Although these studies demonstrated the feasibility of machine learning approaches for MLC positional‐error prediction, they did not account for daily variations in leaf performance throughout a treatment course. Qiu et al. attempted to address this limitation by incorporating MLC‐check parameters to predict the average leaf‐position deviations across control points. Using a random forest algorithm, they predicted the average positional deviations of each leaf across the control points recorded in Dynalog files, achieving an MSE of 0.059 mm^2^ for validation and 0.074 mm^2^ for test datasets. Their results demonstrated that deviations in delivered leaf positions were associated with dosimetric deviations and revealed a substantial correlation between leaf positional deviations and both GPRs and dose deviations, with correlation coefficients ranging from −0.506 to −0.720 for GPRs and from < 0.498 for dose deviations, respectively.^46^ Despite the advantages of the proposed method, as they predicted average leaf positional errors, predicted deviations cannot be integrated into RT‐Plan files to calculate the dose for different structures. The present model addresses this limitation by predicting daily MLC positional discrepancies at the individual leaf and control‐point levels, allowing the predicted leaf positions to be incorporated into the RT‐Plan and subsequently used for daily dose calculation and structure‐specific dosimetric evaluation.

The lower MAE in Lay et al.’s results can be attributed to their larger dataset and lower leaf positional deviations (0.13 mm for total differences in test datasets). Similarly, Osman et al.’s favorable outcomes can be associated with a maximum deviation of 1.2 mm. Though Qiu et al. reported a maximum deviation of 2.21 mm, comparable to the ∼3 mm maximum deviation at the upper whisker limit (Q3 + 1.5×IQR) observed in our dataset, our model still achieved an MSE of 0.020 mm^2^ for the test dataset. The differences between studies may also arise from variations in linear accelerators, MLC models, ML algorithms, dataset size, feature definitions, error distributions, and treatment modalities.

Feature importance analysis from our model highlighted the critical role of initialization parameters in capturing daily discrepancies, with the maximum raw secondary as the most crucial initialization feature. In contrast, Qiu et al. identified PWM as the most correlated feature in their study, which may be attributed to differences in feature contributions during training with different models. Previous studies have demonstrated a linear correlation between individual leaf velocity and the magnitude of positional errors.^59^, ^60^, ^61^ Consistently, our findings identified leaf velocity as the most influential feature during model training, supported by the highest Spearman correlation coefficient. Additionally, the notable contribution of aperture‐based features to loss function reduction reinforces the impact of plan complexity on positional errors.

Analysis of central leaves, as shown in Figure 3, revealed greater positional errors compared to peripheral leaves. This can be attributed to their critical role in intensity modulation and the creation of small apertures, which increase positional uncertainties.

The model demonstrated robust performance on the test dataset, with a maximum absolute positional discrepancy of 3.60 mm between the model predictions and the delivered MLC leaf positions, occurring at a single control point in a brain case. Across the entire test dataset, only 0.02% of the samples showed an absolute prediction error greater than 2 mm, whereas 32% showed an absolute prediction error between 1 and 2 mm. The model also yielded accurate predictions for clinical data spanning six consecutive treatment days, achieving an MAE ranging from 0.051 mm to 0.056 mm and an RMSE between 0.144 mm and 0.150 mm for nine IMRT patients, underscoring its consistent precision in forecasting daily positional errors.

As illustrated in Figure 4, the DVHs consistently showed a trend toward target underdosage, particularly in the steep dose‐gradient region. For OARs, both increases and decreases in dose were observed. For example, in the head and neck case, the planned mean dose for the right parotid was already elevated (30.160 Gy), likely due to competing PTV coverage priorities, whereas the recommended D_mean_ constraint for single gland is < 20 Gy.^62^ An additional increase of approximately 1 Gy could further exacerbate parotid dysfunction.

Furthermore, dosimetric analysis derived from DVHs for the first treatment fraction of nine IMRT plans demonstrated the impact of leaf positional discrepancies on dose distributions, consistent with findings in previous studies.^63^, ^64^, ^65^, ^66^, ^67^, ^68^, ^69^, ^70^ For instance, Bai et al. observed maximum deviations of 4.63%, 8.69%, and 18.32% in the mean dose to the parotid glands with systematic MLC errors of 0.5 mm, 1 mm, and 2 mm, respectively. A 2 mm error in leaf positioning could cause an increase of 0.88%‐1.9% in PTV.^65^

The predominance of negative deviations in the target dose metrics shown in Figure 5 indicates lower dose coverage during delivery in comparison to the planned expectations. Dose reduction can decrease the tumor control probability (TCP), while excessive radiation to OARs may increase the risk of toxicity, particularly in tissues with limited repair capacity. The findings summarized in Table 2 indicate that the average differences in dosimetric indices, except for V_100%_, are not clinically noticeable. For instance, the mean dose reduction for the delivered plan (–0.350 Gy) and the predicted plan (–0.274 Gy) represents only a slight decrease in target irradiation compared to the primary plan. However, in individual cases, the observed minimum and maximum deviations in D_98%_ and D_95%_ indicate that reductions can compromise target coverage, whereas increases may affect nearby OARs. These findings underscore the importance of careful, case‐by‐case evaluation of dosimetric discrepancies.

As shown in Figure 6, the P95 of the absolute MLC positional errors was generally higher for the head and neck plans than for the prostate and brain plans. In the present dataset, the head and neck plans also exhibited higher mean leaf velocities, which may reflect their greater modulation and dynamic leaf‐motion requirements and suggest a potential relationship between treatment‐plan complexity and MLC delivery performance. However, as shown in Figure 7, no clear correlation was observed between the P95 and the dosimetric parameters most affected by MLC positional deviations, namely V_100%_ and D_95%_. This finding suggests that the dosimetric impact of MLC positional deviations is not determined solely by their magnitude but may also depend on their spatial distribution, the local dose gradients, and the anatomical structures affected. For example, prostate case 2 exhibited a substantial reduction in V_100%_ (83%) and an approximately 0.7 Gy change in D_95%_ of the CTV corresponding to the dose fall‐off region of the DVH curve, despite having a P95 of only 0.6 mm. Therefore, the present results suggest that a single threshold based solely on the magnitude of MLC positional error may not adequately represent the clinical importance of the resulting dosimetric changes.

As shown in Figure 9, the maximum positional deviations of delivered MLC leaf positions from their planned positions reached up to 7.33 mm, while the inter‐fraction variations were generally small. The cumulative effect of these daily positional fluctuations is illustrated in Figure 11, which shows the variations in V_100%_ associated with the predicted and delivered leaf positional deviations over six consecutive treatment fractions. These results demonstrate that small fraction‐to‐fraction variations can contribute to observable differences in the delivered dose distribution. The model's limitation in predicting inter‐fraction changes is also reflected in the variation range of V_100%_, which was approximately 2% for the predicted values compared with 4% for the delivered values. In parallel, the most influential initialization feature, maximum raw secondary, also exhibited fluctuations across treatment days (Figure 10), suggesting that changes in daily MLC initialization conditions may contribute to the observed variations in positional errors.

Daily analysis of dose distribution is essential to adaptive radiotherapy planning, which can be modified to account for observed deviations. The advantages of the proposed daily process include improvement of efficiency, reduction of staff workload, plan verification on the treatment day, the ability to analyze dosimetric parameters, and the assessment of the impact of the Linac's daily performance on patient dose. Regarding expected MLC positional deviations and their potential dosimetric consequences before or during a course of treatment, a potential clinical workflow would involve establishing a dosimetric baseline for each individual treatment plan and subsequently evaluating the predicted MLC‐related dose deviations during treatment. If the predicted dose distribution remains within clinically acceptable limits established by the institution and treating physician, treatment could proceed according to the existing QA protocol. Conversely, an unacceptable predicted dosimetric deviation could trigger additional evaluation, such as repeat PSQA, treatment‐plan review, or investigation of MLC performance. This approach could also provide an early warning of potential leaf‐specific performance changes that may warrant further evaluation, calibration, or Periodic Machine Inspection (PMI).

Importantly, the proposed approach is intended to provide plan‐specific surveillance rather than replace established patient‐specific and machine QA procedures. Accurate MLC calibration and robust QA processes remain essential for reliable IMRT delivery, with a 1‐mm tolerance for MLC leaf positioning recommended by AAPM Task Group 142 and Budgell et al,^71^, ^72^ which is also used for Picket Fence test analysis at our institution. However, predefined test patterns used in conventional MLC QA may not fully characterize the plan‐specific dosimetric consequences of MLC deviations during highly modulated IMRT delivery. The proposed approach therefore evaluates MLC positional behavior and its potential dosimetric consequences within the context of the actual treatment geometry, providing information that may not be captured by standardized QA tests alone.

The high GPRs observed across the four criteria (1%/1 mm, 2%/2 mm, 3%/2 mm, and 3%/3 mm) are consistent with the findings of Benjamin et al., who reported a weak correlation between the gamma index and dosimetric errors in patient anatomies.^12^ These findings highlight a limitation of relying solely on gamma analysis, as a high GPR may not necessarily reflect the magnitude or spatial distribution of dose deviations within specific patient structures. In the present study, the measurement‐based PSQA GPRs were slightly lower, ranging from 97.00% to 100% under the 3%/3 mm criterion, which may be partly attributable to additional measurement and procedural uncertainties associated with phantom‐based verification. Therefore, structure‐specific dosimetric evaluation using the proposed ML model may provide additional insight into clinically relevant dose deviations that are not readily apparent from GPR alone.

Several limitations should be considered. First, the study was performed using a limited number of IMRT plans from a single treatment system and MLC configuration which were collected over a relatively short period of time. The model is therefore preliminary, institution‐specific and machine‐specific, and its performance may not generalize to other treatment planning systems, dose‐calculation algorithms, linear accelerators, MLC models, or treatment modalities. External validation using larger, multi‐institutional datasets is required.

Second, although daily initialization parameters were incorporated into the model, their relatively low feature importance suggests that the available measurements may not fully capture fraction‐to‐fraction variations in MLC behavior, potentially limiting the model's sensitivity to subtle temporal changes in MLC performance. To address this, it is recommended that additional daily quality control parameters be incorporated into the prediction models. For example, geometrical and beam evaluations during the daily Machine Performance Check (MPC) of Varian TrueBeam machines can serve as more comprehensive daily quality control features. Nevertheless, these findings suggest that daily initialization measurements provide useful information but may not capture all transient, stochastic, or abrupt changes in MLC behavior. Consequently, the proposed model should not be regarded as a standalone machine‐failure detection system. Considering the varying complexity and delivery characteristics of different treatment plans, treatment‐site‐specific models may potentially improve predictive performance. Future research should also investigate integration of the model into dose calculation workflows and evaluate the clinical and radiobiological consequences of dose discrepancies, particularly in hypofractionated treatments. Prospective studies involving larger datasets, systematically varied treatment complexity, multiple MLC and accelerator platforms, and documented changes in machine performance are required before the proposed approach can be considered for routine clinical implementation.

## 5 CONCLUSION

In this study, we developed a predictive model to estimate daily MLC positional discrepancies and assess corresponding daily dose‐volume histograms (DVHs) and dosimetric indices. By incorporating MLC daily check parameters alongside aperture‐based and mechanical leaf parameters, the proposed LightGBM model demonstrated robust performance, achieving an MAE of 0.054 mm, an RMSE of 0.144 mm and an R^2^ score of 0.932 for the test dataset. The model's high accuracy in predicting dosimetric indices and daily DVHs further validated its precision. While notable variations in dosimetric parameters were observed due to MLC positional errors, the GPR analysis especially using the 3%/3 mm gamma index, confirmed the feasibility of treatment plan delivery. Therefore, the proposed approach may serve as a plan‐specific surveillance tool, rather than replacing established patient‐specific and machine QA procedures, and could provide a basis for further investigation of its potential role in future adaptive radiotherapy workflows.

## AUTHOR CONTRIBUTIONS

Samira Aghaei Akizi led the programming, experiments, analysis, and manuscript writing. Dr. Zahra Siavshpour contributed to the study design and provided valuable support in analysis and writing. Sohrab Barimani assisted with the recording and access of Dynalog files on the Clinac system. Dr. Seyed Mahmoud Reza Aghamiri and Dr. Reza Ghaderi offered insightful guidance and feedback during the manuscript revision.

## CONFLICT OF INTEREST STATEMENT

The authors declare no conflicts of interest.

## FUNDING INFORMATION

The authors have nothing to report.

## ETHICS APPROVAL

This retrospective study was approved by the Ethics Committee of Shahid Beheshti University of Medical Science (IR.SBMU.RETECH.REC.1404.371).

## CONSENT FOR PUBLICATION

Not applicable

## CONSENT TO PARTICIPATE

According to the approved protocol, informed consent to participate was waived. The study was conducted in accordance with the principles of the Declaration of Helsinki.

## Supporting information




**Supporting Information**: acm270882‐sup‐0001‐SupMat‐S01.docx

**Supporting Information**: acm270882‐sup‐0002‐SupMat‐S02.docx

**Supporting Information**: acm270882‐sup‐0003‐SupMat‐S03.docx

**Supporting Information**: acm270882‐sup‐0004‐SupMat‐S04.docx

**Supporting Information**: acm270882‐sup‐0005‐SupMat‐S05.jpg

## Data Availability

Data is available as supplemental files associated with the online journal article.
