# Supplementary material for: Daily Multi‐Leaf Collimator positional error prediction using machine learning: A Dynalog‐based approach for patient specific quality assurance
Source: J Appl Clin Med Phys. 2026 Oct 27;27(11):e70882. doi: 10.1002/acm2.70882 (PMC13714354; doi:10.1002/acm2.70882)
Supplement: Supplementary file 1 [file ACM2-27-e70882-s001.docx]

Table S1. The values considered for each hyperparameter of the LightGBM model.

| Hyperparameter | Candidate values | Final value |
| --- | --- | --- |
| num_leaves | 31, 51, 100 | 51 |
| num_iterations | 50, 100, 200, 300, 400, 500 | 500 |
| learning_rate | 0.01, 0.05, 0.10, 0.20 | 0.05 |
| max_depth | −1 | −1^1^ |
| min_data_in_leaf | 1, 20, 50, 120 | 120 |
| lambda_l1 | 0.0, 0.1, 0.5, 1.0 | 0.10 |
| lambda_l2 | 0.0, 0.5, 1.0, 1.01, 1.02, 1.03, 1.05, 2.0 | 1.03 |
| bagging_fraction | 0.5, 0.8, 1.0 | 1.0 |
| feature_fraction | 0.5, 0.8, 1.0 | 1.0 |
| bagging_freq | 0, 1 | 0 |
| boosting_type | GBDT | GBDT |
| objective | Regression | Regression |
| random_seed | Fixed at 42 | 42 |
| ^1^ In accordance with the leaf-wise tree growth strategy of LightGBM, max_depth was fixed at −1, and num_leaves was optimized as the primary parameter controlling tree complexity. | | |
