# Supplementary material for: Daily Multi‐Leaf Collimator positional error prediction using machine learning: A Dynalog‐based approach for patient specific quality assurance
Source: J Appl Clin Med Phys. 2026 Oct 27;27(11):e70882. doi: 10.1002/acm2.70882 (PMC13714354; doi:10.1002/acm2.70882)
Supplement: Supplementary file 2 [file ACM2-27-e70882-s002.docx]

**Figure 1.** Learning curves of the LightGBM model with the optimized hyperparameters during three-fold patient/plan-level cross-validation using 78 features, showing MSE for the training and validation datasets as a function of the number of boosting iterations. Shaded regions represent ±1 standard deviation across the three cross-validation folds. The vertical dashed line indicates the selected 500 boosting iterations.

Figure 2. Model performance assessed using (a) correlation analysis and (b) Bland–Altman analysis between predicted and delivered leaf positional errors for test dataset. The dashed black line in (a) represents the line of best fit, with a coefficient of determination (R²) of 0.932. In (b), the dashed red line indicates the mean bias, and the dashed black lines denote the 95% limits of agreement.

Figure 3. Differences between the planned and delivered (a), and predicted and delivered (b) leaf positions across all control points in the test dataset. For clarity, each subplot is divided into a bottom panel (leaves 1–40) and a top panel (leaves 41–80). Each box represents the distribution of positional errors for an individual leaf, with the orange and green horizontal lines indicating the median and mean values, respectively. The boxes extend from the first quartile (Q1) to the third quartile (Q3), while the whiskers extend to Q1 − 1.5 × IQR and Q3 + 1.5 × IQR, where IQR denotes the interquartile range. Data points falling outside this range represent larger positional errors and are shown as blue dots.

Figure 4. DVH curves derived from dose distributions corresponding to the planned, delivered, and predicted MLC positions of first treatment fraction and selected OARs and PTVs for three treatment sites: brain, prostate, and head and neck. Solid lines represent the planned, dashed lines correspond to the delivered, and dash-dotted lines indicate the predicted. The vertical black dashed line indicates the prescribed dose for each case.

Figure 5. Distribution of dosimetric index deviations from the planned values for the dose distributions obtained from the delivered and predicted MLC leaf positions for target and OAR structures across nine IMRT plans for the first treatment fraction. The upper (blue) and lower (brown) lobes of the violin plots represent the deviations associated with the delivered and predicted MLC leaf positions, respectively. Dark vertical lines within each plot, along with scatter points (green circles and orange crosses) between the lobes, are included for enhanced visualization.

Figure 6. Comparison of the P95 of the delivered and predicted absolute leaf positional error averaged over six consecutive treatment days for 9 IMRT plans of different cases. Circles indicate the delivered values and crosses indicate the predicted values. The mean leaf velocity is shown for each treatment plan, and the colors of the case labels indicate the treatment site (head and neck, prostate, and brain).

Figure 7. Dosimetric parameter differences averaged across treatment fractions extracted from dose distributions of delivered and predicted leaf positions relative to the plan (a), and correlations between the 95th percentile of the absolute MLC positional errors and the most deviated PTV dosimetric parameters, V_100%_ and D_95%_ of delivered dose distribution for each case relative to plan (b).

**Figure 8.** Top 10 OAR structures showing the largest absolute deviations in dosimetric parameters between the delivered and planned, and predicted and planned dose distributions, averaged over six consecutive treatment fractions across the nine IMRT cases.

**Figure 9. Comparison of the maximum delivered and predicted positional deviations of individual MLC leaves from their planned positions across six consecutive treatment fractions of a head and neck IMRT plan. The leaf exhibiting the maximum delivered positional deviation in each MLC bank is indicated by a green arrow. For clarity, leaves 1–40 are shown at the bottom panel, and the top panel shows leaves 41–80.**

Figure 10. Variations of max raw secondary relative to the first treatment day, averaged across six consecutive treatment days for each leaf (a), variation of maximum raw secondary readout averaged across all leaves at each treatment day (b). The bottom panel shows leaves 1–40, and the top panel shows leaves 41–80.

Figure 11. Variation in the V_100%_ of PTV across six consecutive treatment days for a prostate patient with the largest observed deviation in this parameter. The difference between the delivered maximum and minimum V_100%_ are represented in the light blue region.
