# Supplementary material for: Daily Multi‐Leaf Collimator positional error prediction using machine learning: A Dynalog‐based approach for patient specific quality assurance
Source: J Appl Clin Med Phys. 2026 Oct 27;27(11):e70882. doi: 10.1002/acm2.70882 (PMC13714354; doi:10.1002/acm2.70882)
Supplement: Supplementary file 3 [file ACM2-27-e70882-s003.docx]

Table S2. The 25 most important features identified by LightGBM, accounting for 98% of the total feature importance, ranked in descending order of importance, with their feature categories and Spearman correlation coefficients with MLC positional discrepancies.

|  | Selected feature (LightGBM) | Feature category | Spearman correlation coefficient |
| --- | --- | --- | --- |
| 1 | **Leaf velocity** | **Mechanical** | **0.703** |
| 2 | **MAD_cp_** | **Aperture-based** | **-0.359** |
| 3 | Leaf position | **Mechanical** | 0.335 |
| 4 | MAD_beam_ | Aperture-based | -0.334 |
| 5 | Right border of leaf | **Mechanical** | -0.374 |
| 6 | Left border of leaf | **Mechanical** | -0.326 |
| 7 | Leaf pair gap | Aperture-based | 0.597 |
| 8 | SAS below 2mm | Aperture-based | -0.590 |
| 9 | Skewness of Leaf-Pair Gap Distribution | Aperture-based | 0.001 |
| 10 | Leaf acceleration | **Mechanical** | 0.050 |
| 11 | Young complexity score | Aperture-based | 0.003 |
| 12 | LSV | Aperture-based | 0.113 |
| 13 | leaf movement direction | **Mechanical** | -0.638 |
| 14 | Number of control points per beam | **Mechanical** | 0.028 |
| 15 | **Maximum raw secondary** | **Initialization** | **0.431** |
| 16 | CAS | Aperture-based | -0.009 |
| 17 | MCS_cp_ | Aperture-based | 0.126 |
| 18 | Gantry angle | **Mechanical** | 0.040 |
| 19 | MCS_beam_ | Aperture-based | 0.113 |
| 20 | MU | **Mechanical** | 0.007 |
| 21 | BA | Aperture-based | 0.090 |
| 22 | Leaf-Pair Gap Categories below 2mm | Aperture-based | -0.159 |
| 23 | SAS below 2mm | Aperture-based | -0.588 |
| 24 | Leaf Perimeter-to-Aperture Area Ratio | Aperture-based | 0.002 |
| 25 | CIAO area | Aperture-based | 0.145 |
