# Supplementary material for: Daily Multi‐Leaf Collimator positional error prediction using machine learning: A Dynalog‐based approach for patient specific quality assurance
Source: J Appl Clin Med Phys. 2026 Oct 27;27(11):e70882. doi: 10.1002/acm2.70882 (PMC13714354; doi:10.1002/acm2.70882)
Supplement: Supplementary file 4 [file ACM2-27-e70882-s004.docx]

Figure S1. DVH curves derived from dose distributions corresponding to the planned, delivered, and predicted MLC positions across six consecutive treatment fractions and selected OARs and PTVs for three treatment sites: prostate, brain, and head and neck. Solid lines represent the planned, dashed lines correspond to the delivered, and dash-dotted lines indicate the predicted. The vertical black dashed line indicates the prescribed dose for each case.
